# Comprehensive isotopomer analysis of glutamate and aspartate in small tissue samples

**DOI:** 10.1016/j.cmet.2023.07.013

**Published:** 2023-08-22

**Authors:** Feng Cai, Divya Bezwada, Ling Cai, Rohit Mahar, Zheng Wu, Mario C. Chang, Panayotis Pachnis, Chendong Yang, Sherwin Kelekar, Wen Gu, Bailey Brooks, Bookyung Ko, Hieu S. Vu, Thomas P. Mathews, Lauren G. Zacharias, Misty Martin-Sandoval, Duyen Do, K. Celeste Oaxaca, Eunsook S. Jin, Vitaly Margulis, Craig R. Malloy, Matthew E. Merritt, Ralph J. DeBerardinis

**Affiliations:** 1 Children’s Medical Center Research Institute, University of Texas Southwestern, Medical Center, Dallas, TX, 75390 USA; 2 Quantitative Biomedical Research Center, Department of Population and Data Sciences, UT Southwestern Medical Center, Dallas, TX, 75390 USA.; 3 Simmons Comprehensive Cancer Center, UT Southwestern Medical Center, Dallas, TX, 75390 USA.; 4 Department of Biochemistry and Molecular Biology, University of Florida, Gainesville, FL, 32603 USA; 5 Advanced Imaging Research Center, University of Texas Southwestern Medical Center, Dallas, TX, 75390 USA; 6 Department of Urology, University of Texas Southwestern Medical Center, Dallas, TX, 75390 USA; 7 Department of Internal Medicine, University of Texas Southwestern Medical Center, Dallas, TX, 75390 USA; 8 Department of Radiology, University of Texas Southwestern Medical Center, Dallas, TX, 75390 USA; 9 Veterans Affairs North Texas Healthcare System, Dallas, TX, 75216 USA; 10 Howard Hughes Medical Institute, University of Texas Southwestern Medical Center, Dallas, TX, 75390 USA; 11 Lead Contact

## Abstract

Stable isotopes are powerful tools to assess metabolism. ^13^C labeling is detected using nuclear magnetic resonance (NMR) spectroscopy or mass spectrometry (MS). MS has excellent sensitivity but generally cannot discriminate among different ^13^C positions (isotopomers), whereas NMR is less sensitive but reports some isotopomers. Here, we develop an MS method that reports all 16 aspartate and 32 glutamate isotopomers while requiring less than 1% of the sample used for NMR. This method discriminates between pathways that result in the same number of ^13^C labels in aspartate and glutamate, providing enhanced specificity over conventional MS. We demonstrate regional metabolic heterogeneity within human tumors, document the impact of fumarate hydratase deficiency in human renal cancers, and investigate the contributions of TCA cycle turnover and CO_2_ recycling to isotope labeling in vivo. This method can accompany NMR or standard MS to provide outstanding sensitivity in isotope labeling experiments, particularly in vivo.

## eTOC

Cai et al. developed a sensitive mass spectrometry method to report all 16 aspartate and 32 glutamate positional ^13^C isotopomers. In small tissue samples labeled with ^13^C, the method reveals aspects of TCA cycle metabolism difficult or impossible to detect with non-positional mass spectrometry or NMR.

## Graphical abstract

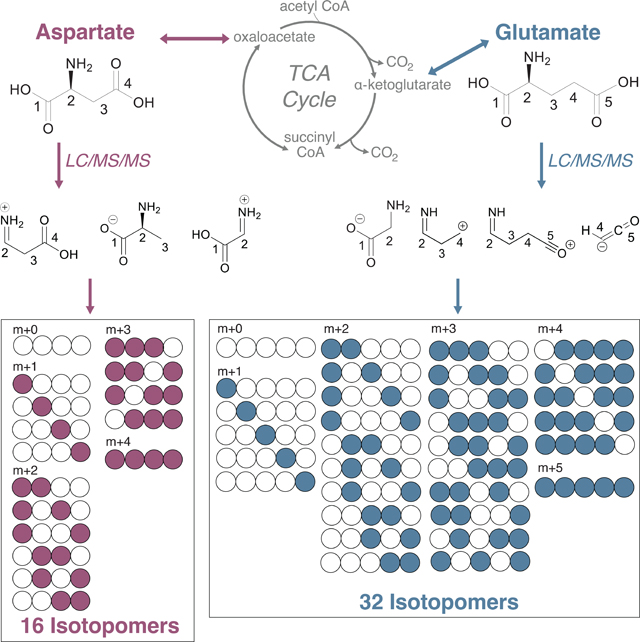

## INTRODUCTION

Stable isotope tracers including ^13^C, ^2^H and ^15^N are powerful tools to probe metabolism^1,2^. Because these isotopes do not undergo radioactive decay, they are considered safe to administer and a wide variety of labeled nutrients are readily available for experimental or clinical use. Nutrients labeled with a stable isotope (e.g. ^13^C-glucose) may be introduced to the study subject via enteral or parenteral routes. Metabolism of the labeled nutrient transmits the isotope to metabolic products. Labeling in these products, obtained through fluid or tissue sampling, can be inspected to infer metabolic activity in the intact system. In humans, stable isotope tracing has been used to assess turnover of glucose, lipids, and proteins; rates of whole-body substrate oxidation; and alteration of these processes by disease^3–7^. Recent studies have assessed human tumor metabolism by examining isotope transfer from labeled nutrients in the circulation to metabolites extracted from tumor specimens^8–13^.

Mass spectrometry (MS) and nuclear magnetic resonance (NMR) spectroscopy can measure isotope enrichment in metabolites of interest. MS has excellent sensitivity and determines both the total enrichment of a metabolite pool and the contributions of each isotopologue to the pool. For example, in a ^13^C labeling experiment designed to examine a metabolite with *i* carbons, MS reports the fraction of the pool contributed by M+0, M+1, M+2 … M+*i* forms of the metabolite, where M is the mass of the unlabeled metabolite. Assessment of isotopologues by MS does not report the position of ^13^C within the metabolite. This is a significant limitation, because a metabolite of *i* carbons has *i*+1 isotopologues but 2^*i*^ labeled forms (isotopomers) when the position of each ^13^C is considered. Therefore, isotopologue analysis by simple MS is informative but lacks the full complement of information afforded by isotopomer analysis. NMR is the analytical method of choice for isotopomer analysis because the local magnetic environment around an atomic nucleus translates into a predictable position on a chemical shift spectrum, which allows the position of ^13^C within a molecule to be reported^14^. However, NMR generally does not report all possible isotopomers and it has low sensitivity relative to MS.

Isotopomer analysis has classically been used to assess the tricarboxylic acid (TCA) cycle, a mitochondrial pathway of fuel oxidation^15^. This pathway is central to both energy formation and biosynthesis because it provides reducing equivalents for oxidative phosphorylation and intermediates for lipid, protein, and nucleotide biosynthesis. Carbon enters the TCA cycle via acetyl-CoA, which largely arises from pyruvate dehydrogenase (PDH) and fatty acid or ketone oxidation; or through anaplerotic pathways supplied by pyruvate carboxylation and oxidation of some amino acids and other fuels. The relative contributions of different acetyl-CoA sources, anaplerosis, pyruvate recycling and cycle turnover are encoded by specific ^13^C isotopomers in metabolites related to the TCA cycle. Glutamate isotopomers are particularly useful because glutamate provides information about the acetyl-CoA and oxaloacetate (OAA) that supply citrate synthase, and because the high intracellular abundance of glutamate makes it a convenient metabolite for analysis.

Previous studies have used fragmentation patterns in tandem mass spectrometry to examine isotopomers of metabolites related to the TCA cycle. Gas chromatography-tandem mass spectrometry (GC-MS/MS) was used to partially resolve isotopomers of glutamate extracted from hearts perfused with ^13^C-labeled fuels^16^. GC-MS/MS was also used to derive all 16 aspartate isotopomers^17^_._ Either a combination of LC-MS and NMR or LC-MS/MS alone was used to study bacterial samples containing OAA isotopomers^18^ or mixed standards of malate isotopomers^19^. Grouped rather than individual isotopomers from multiple TCA cycle intermediates, including some groups of glutamate isotopomers, were determined with liquid chromatography-tandem mass spectrometry (LC-MS/MS) and used to calculate fluxes in cultured cells^20^.

We set out to develop an LC-MS/MS method to identify the 32 individual isotopomers of glutamate and 16 individual isotopomers of aspartate. Our motivation is the increasing use of stable isotope tracers to assess cancer metabolism in vivo. These studies produce complex labeling patterns that are difficult to interpret without positional assignment of ^13^C. The large sample size required for NMR makes it impractical to use this technique to study regional heterogeneity in ^13^C labeling, which is significant in solid tumors. We benchmarked the new method against NMR and find that it provides similar information about isotopomer distributions despite requiring only 16,000 cells or 0.5 mg of tissue. We also demonstrate the method’s superiority to simple isotopologue analysis in assessing metabolism of cultured cancer cells and tumors growing in mice and patients.

## DESIGN

**Figure 1.**
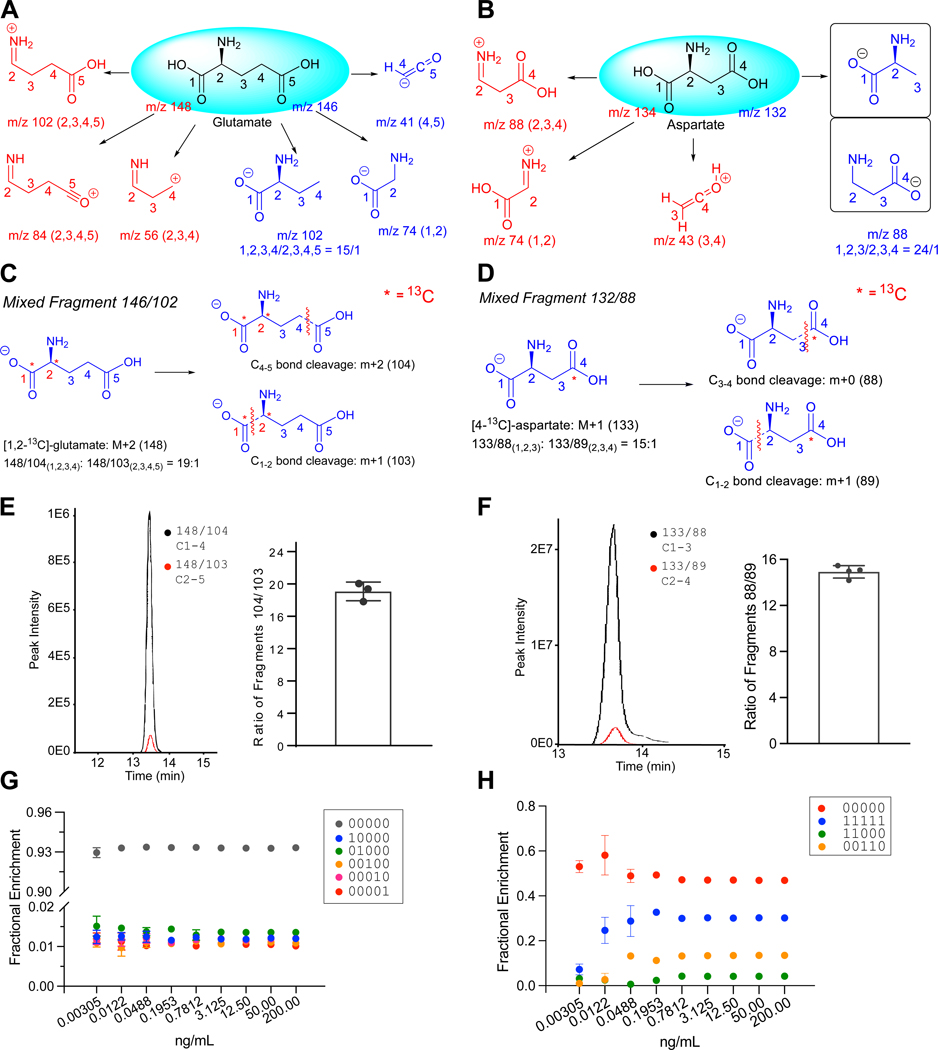
Development of LC-MS/MS analysis of glutamate and aspartate isotopomers. (A-B) LC-MS/MS fragmentation of glutamate (A) and aspartate (B). Carbon numbers correspond to positions in the unfragmented molecule. Precursor/product ion pairs are described in the text. (C) Composition of the mixed 146/102 fragment from glutamate. (D) Composition of the mixed 132/88 fragment from aspartate. (E) Relative abundance of the 148/104 (C1–4) and 148/103 (C2–5) ion pairs from a [1,2-^13^C]glutamate standard (3 technical replicates). (F) Relative abundance of the 133/88 (C1–3) and 133/89 (C2–4) ion pairs from a [4-^13^C]aspartate standard (4 technical replicates). (G) Relative quantitation of naturally-occurring glutamate isotopomers (3 technical replicates). (H) Relative quantitation of isotopomers from a mixture of [1,2-^13^C]glutamate (5%); [3,4-^13^C]glutamate (15%); [U-^13^C]glutamate (30%); and unlabeled glutamate (50%), across a range of concentrations (3 technical replicates). The data in panels E-H are shown as mean ± standard deviation.

We first developed an approach to detect all 32 glutamate isotopomers using multiple reaction monitoring (MRM) with LC-MS/MS. Glutamate contains five carbons and four carbon-carbon σ bonds. We examined the fragmentation pattern of glutamate and found seven precursor/product ion pairs (M/m) as summarized in Fig.1A, in which precursor ions are denoted with a capital M and product ions are denoted with a lowercase m. Two ion pairs, 146/41 and 146/59, report the C4–5 fragment, but the 146/41 ion pair produces a higher-quality peak on a Sciex QTRAP 6500 and was used in the analyses below. The 146/74 and 148/84 (or 148/102) ions report the C1–2 and C2–5 fragments, respectively. Both 148/84 and 148/102 positive ions resulted from C1-C2 cleavage and showed high quality peaks, but 148/84 was chosen because it provided more consistent signals. The 146/102 negative ion is a mixture of C1–4 and C2–5 fragments, both resulting from CO_2_ loss during fragmentation. By examining the 148/104 and 148/103 ions fragmented from a [1,2-^13^C]glutamate standard (denoted as glutamate 11000 throughout this paper, with 1 indicating ^13^C and 0 indicating ^12^C), we determined that the C1–4 and C2–5 fragments are generated at a fixed ratio of 19:1, allowing relative quantitation of these two fragments (Fig. 1C, E)^19^. The 148/56 ion mainly arises from the C2–4 fragment along with a negligible amount (2%) of C3–5. These fragments were validated by examining the 150/57 and 150/56 fragmentation of a glutamate 11000 standard (Supplementary Data I, Scheme 1a).

It is worth noting that the 146/41 ion pair can also be generated from the C2–4 fragment (C_3_H_5_^-^) and may interfere with the C4–5 fragment (C_2_HO^-^)^19^. The contribution of C2–4 versus C4–5 in the 146/41 ion pair can be distinguished by measuring the 151/43 (M+5/m+2, ^13^C_2_HO^-^, C4–5 fragment) and 151/44 (M+5/m+3, ^13^C_3_H_5_^-^, C2–4 fragment) ion pairs from a glutamate 11111 standard (Supplementary Data I, Scheme 1b). We observed both 151/43 and 151/44 on a Sciex QTRAP 5500, but the 151/44 ion pair was below the detection limit on a Sciex QTRAP 6500. If both ion pairs are detected, their ratio should be considered in the isotopomer distribution matrix.

With these five main precursor/product ion pairs (146/41, 146/74, 146/102, 148/56, and 148/84) in unlabeled glutamate, additional ion pairs can differentiate labeled higher order isotopomers in the format M+*i*/m+*j* (*j*≤*i*), in which *i* denotes the number of ^13^C in the precursor ion and *j* denotes the number of ^13^C in the product ion. Taking all the ^13^C isotopomers into account, 88 total ion pairs were identified (Supplementary Data II). There are twenty ion pairs associated with labeled forms of 146/102, which mainly represent the C1–4 fragment, and 148/56, which includes the C2–4 fragment and therefore also reports information about C4-C5 bond cleavage. These twenty ion pairs seem redundant, but this complementary information can improve accuracy in some samples. For example, the chromatogram peak for 147/75, an M+1/m+1 labeled form of 146/74, often overlaps with another metabolite. Although this prevents precise analysis of the 147/75 ion pair, other ion pairs provide complementary information (Supplementary Data I).

We performed a similar isotopomer analysis of aspartate (Fig. 1B). We identified 4 ion pairs (134/88, 134/74, 134/43 and 132/88) to distinguish the C-C bond cleavage site. The 132/88 ion pair is a mixture of fragments C1–3 and C2–4 (Fig. 1D). Using an aspartate 0001 standard, we measured the ratio of 133/88 and 133/89 to differentiate the contribution of C1–3 versus C2–4 in ion pair 132/88 and determined that the average ratio of C1–3:C2–4 is 15:1 (Fig. 1F). There are twelve ion pairs associated with labeled forms of 134/43 and these ion pairs provide redundant information to improve accuracy.

The distribution matrix that linearly maps the 88 ion pairs to all 32 glutamate isotopomers only has a rank of 28 out of a full rank of 32. Obtaining additional fragments of C2–3 and C3–4 may produce a complete rank, but this is not feasible due to the chemical properties of glutamate. Gratifyingly, nonnegative least square regression fit in this situation provides information about all 32 glutamate isotopomers. The fitting results were then evaluated through a survey of 5,000 groups of 32 random fractions using the R script that was uploaded in GitHub. The result is shown in Supplemental Data I, Figure SD-I 1, which includes the median and 95% confidence interval for isotopomer distribution error based on this simulation. Eighteen of the glutamate isotopomers are highly precise with absolute errors less than 7e-17. The remaining fourteen have absolute errors up to +/−4%. In reality, a single tracer would predominantly generate a major isotopomer of acetyl-CoA such as [1-^13^C], [2-^13^C], or [1,2-^13^C]acetyl-CoA. After incorporating this information, we conducted new surveys with the contents of two acetyl-CoA isotopomers summing to no more than 2% of the third isotopomer and found that the highest error in the 14 less accurate isotopomers declined to below 0.5%. Therefore errors can be mitigated by choosing labeling strategies that minimize the contributions of mixed acetylCoA isotopomers.

The 14 isotopomers with the highest apparent uncertainty are all from M+2 or M+3 isotopologues, and 10 of the 14 contain only single labels in their C4–5 fragments. These isotopomers are relatively scarce in tracing experiments that produce [1,2-^13^C]acetyl-CoA (e.g. [U-^13^C]glucose and [1,2-^13^C]acetate). Two additional isotopomers (glutamate 10011 and 01011) are downstream of OAA 0001, 1001, 0010, or 1010). These isotopomers are also expected to be scarce when the major labeled form of acetyl-CoA is [1,2-^13^C]acetyl-CoA (Fig. S1). The last two isotopomers, glutamate 10100 and 01100, provide information related to TCA cycle turnover but have relatively high errors. However, these two isotopomers are directly proportional to glutamate 10111 and 01111 and the fraction of acetyl-CoA supplying the TCA cycle and labeled as [1,2-^13^C]acetyl-CoA (F_c3_, see below). Thus, these last two isotopomers can be evaluated by combining an analysis of glutamate 10111 and 01111 with F_c3_. We performed similar calculations for all 16 aspartate isotopomers and evaluated their accuracy with 16 random fractions generated through beta distributions (Supplementary Data I, Fig. SD-I 2). Four isotopomers, aspartate 1010, 0110, 1001, and 0101, produced errors up to +/− 6%. These four isotopomers are not expected to be abundant in tracing experiments that produce [1,2-^13^C]acetyl-CoA.

To validated the glutamate isotopomer method, we first examined a naturally occurring glutamate standard across a range of concentrations without natural abundance correction (Fig. 1G). This revealed the presence of all five forms of singly-labeled glutamate (at positions C1-C5) at approximately 1% each, close to the expected 1.1% natural abundance of ^13^C at each position. As expected, unlabeled glutamate was approximately 94% (Fig. 1G). The absolute errors are approximately 0.15%, with higher errors of 0.3% for glutamate C2, representing a relative error of 30%. The relative errors for C5 are as low as 10%, making it one of the most accurate positions (Fig. S2A). In this experiment, errors arise in part to the fact that the natural abundance distribution in Fig. 1G is uncorrected. MRM is a low resolution MS method and cannot distinguish between ^15^N and ^13^C. Multiple fragments contain nitrogen, and the C2 and nitrogen are bonded in all related fragments. But in Figure 1G, ^15^N was assigned as the more naturally abundant ^13^C (^15^N is 0.37% natural abundance whereas ^13^C is 1.1%). This artificially increases the fraction of some isotopomers, especially 01000. Correcting for natural abundance isotopes with a corresponding matrix mitigates these small absolute errors across a range of glutamate concentrations, and as a result all singly ^13^C-labeled glutamates including 01000 are essentially zero, while unlabeled glutamate is above 99.9% (Fig. S2B).

We next examined a solution of 50% glutamate 00000, 5% glutamate 11000 (99% isotope purity, 98% chemical purity), 15% glutamate 00110 (99% isotope purity, 98% chemical purity) and 30% glutamate 11111 (99% isotope purity, 98% chemical purity). Different concentrations of this mixture were used to explore the dynamic range of the method (Fig. 1H). In each sample, all 32 isotopomers were analyzed. Our measurements of these isotopomers by LC-MS/MS resulted in absolute errors of 1% or less. In addition, the relative standard deviation (RSD) values were below 5% at a glutamate concentration of 0.78 ng/mL, and below 2% at a glutamate concentration of 3.12 ng/mL. Lower concentrations result in poor signals of some key ion pairs (e.g. 146/41, 146/74). We note that some degree of saturation at the ion source interferes with glutamate ionization but does not affect the ratios among different isotopomers^21,22^. However, saturation of the mass spectrometer detector should be avoided.

## RESULTS

### Validating LC-MS/MS method against NMR isotopomer analysis.

**Figure 2.**
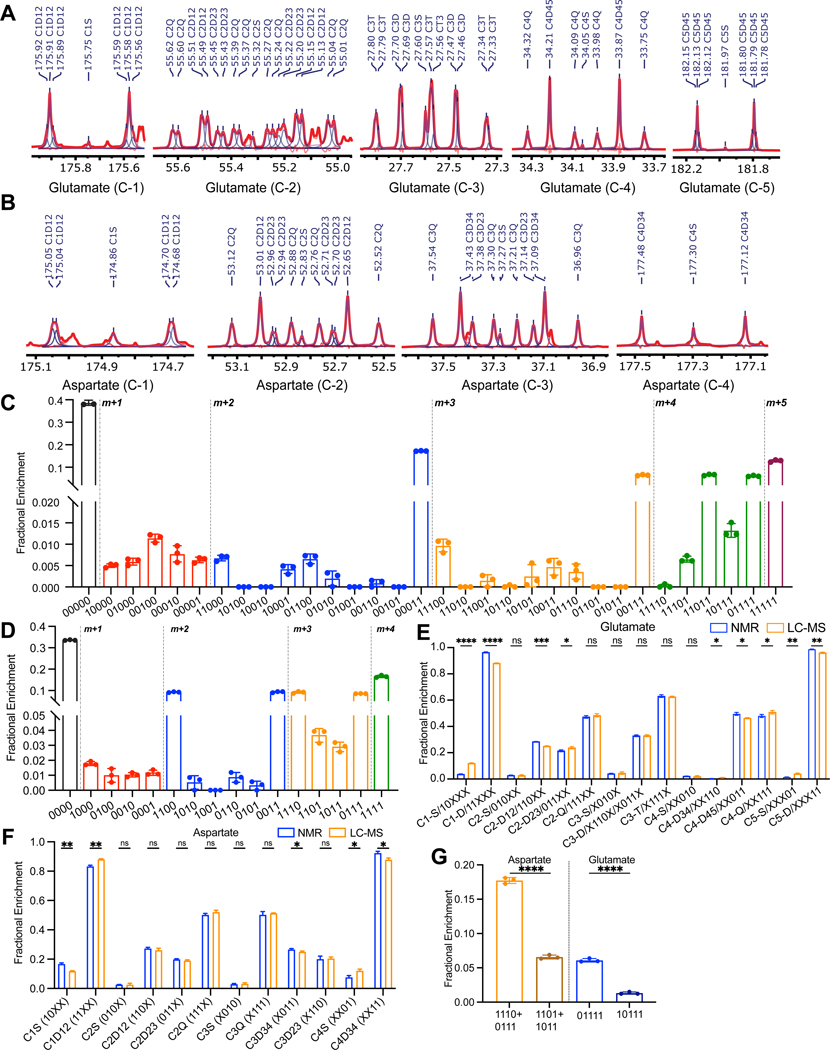
Comparison of isotopomer analysis from NMR and LC-MS/MS. (A) An example of 1D ^13^C NMR spectrum from H460 lung cancer cells cultured with [U-^13^C]glucose. Multiplets from all five glutamate carbons are displayed. (B) 1D ^13^C NMR spectrum from the same culture as shown in (A) displaying multiplets from all four aspartate carbons. (C) Fractional enrichment of 32 glutamate isotopomers from H460 lung cancer cells cultured with [U-^13^C]glucose by LC-MS/MS (3 biological replicates). (D) Fractional enrichment of 16 aspartate isotopomers of H460 lung cancer cells cultured with [U-^13^C]glucose by LC-MS/MS (3 biological replicates). (E) Comparison of glutamate isotopomer analysis by ^13^C NMR and LC-MS/MS using data from (C) (3 biological replicates). (F) Comparison of aspartate isotopomer analysis by ^13^C NMR and LC-MS/MS using data from (D) (3 biological replicates). (G) The sum of aspartate 1110 and 0111, which arise from PC, versus aspartate 1101 plus 1011, which arise from multiple pathways, and the corresponding differences in glutamate 01111 and 10111 (3 biological replicates). The data in panels C-G are shown as mean ± standard deviation. P values: ns = P > 0.05; * =P ≤ 0.05; ** = P ≤ 0.01; *** = P ≤ 0.001; **** = P ≤ 0.0001. Unpaired t tests were used for panels E-G.

To extend the isotopomer method to biological samples and benchmark it against 1D ^13^C NMR, we cultured H460 lung cancer cells in medium containing [U-^13^C]glucose for 24 h and then extracted the metabolites from three 15 cm dishes at approximately 90% confluence. We used 1% of the extract for LC-MS/MS and the rest for NMR. ^13^C NMR reported the relative abundance of a subset of glutamate and aspartate isotopomers based on spin coupling generated by adjacent ^13^C nuclei (Fig. 2A,B), whereas LC-MS/MS analysis reported all 32 isotopomers of glutamate and all 16 isotopomers of aspartate (Fig. 2C,D). To directly compare the isotopomer distributions reported by the two methods, data from LC-MS/MS were left uncorrected for naturally occurring isotopes and summarized in the same format as the ^13^C NMR data (Fig. 2E, F). This revealed excellent consistency for isotopomers related to glutamate carbons C2–4 and aspartate carbons C2–3, which contain most of the information relevant to TCA cycle turnover. Glutamate C1-S/C1-D and C5-S/C5-D exhibit consistent differences between NMR and LC-MS/MS (Fig. 2E, S2C). However, the percentages of both C1-S and C5-S in the NMR data are under 5% and may not be accurate. Therefore, it is difficult to benchmark the LC-MS/MS data from these carbons against NMR. Other differences like glutamate C2-D and aspartate C1-S/C1-D are also apparent but do not follow consistent trends among the samples. The issue with these carbons seems to lie with the NMR analysis. The aspartate C1 singlet sometimes overlaps with resonances from other metabolites, complicating the accurate integration of the C1 multiplet (Fig. S2D). In spectra lacking these overlapping resonances (e.g. Fig, 2B), analysis of aspartate C1 was concordant between NMR and LC-MS/MS (Fig. S2C). Overall, although there are some differences between LC-MS/MS and NMR, they generally involve limitations of NMR related to overlapping signals, which are complicated further by low isotopomer abundance.

An advantage of isotopomer analysis is that it allows the calculation of metabolic parameters such as F_C3._ This term denotes the fraction of acetyl-CoA supplying the TCA cycle with ^13^C at both the carbonyl and methyl carbons. Anaplerosis (y) can also be calculated through isotopomer analysis. Using samples from the [U-^13^C]glucose culture, we compared F_C3_ and y values derived from NMR and LC-MS/MS. From the LC-MS/MS data, F_C3_ was calculated with a formula commonly used in NMR (eq. 1) or by directly using glutamate 11000 and 11011 (eq. 2). These equations calculated F_C3_ as 0.865±0.004 and 0.907±0.012, respectively. Eq. 1 also calculated F_C3_ as 0.898±0.056 from the NMR data. Using an equation developed for NMR (eq. 3), relative anaplerotic flux (y) was calculated as 0.351±0.014 from LC-MS/MS data and 0.436±0.047 from NMR data (data in Supplementary Data II). Therefore, the new LC-MS/MS analysis provides similar information about metabolism as the established NMR approach while requiring much less input material.


(1) $$F_{C3}=GLU4Q\times \frac{GLU4}{GLU3}$$

(2) $$F_{C3}=\frac{glu11011}{glu11000+glu11011}$$

(3) $$y=\frac{\frac{GLU_{4}}{GLU_{3}}-1}{2}$$

Glu4Q is the area of the glutamate C4 quartet divided by the total C4 area, while 30 Glu4/Glu3 is the ratio of total Glu4 resonance to total Glu3 resonance.

It is significant that the fractions of aspartate 1110 and 0111 differ from aspartate 1101 and 1011 (Fig. 2D). In a simple metabolic network with Ac-CoA as the only carbon source for the TCA cycle, these four m+3 isotopologues should be present in equal fractions because of the symmetric structure of succinate and fumarate (Fig. S1A). However, ^13^C enters the TCA cycle as both acetyl-CoA and OAA in cells with concomitant PDH and pyruvate carboxylase (PC) activity (Fig. S1B). The difference between the sum of aspartate 1110 and 0111, which arise initially from PC, and aspartate 1101 plus 1011, which arise from multiple pathways, indicates that PC is active and contributes to the TCA cycle (Fig. 2G). The corresponding difference is found in glutamate, in which glutamate 01111 (arising from oxaloacetate 1110 and [1,2-^13^C]acetyl-CoA) is more abundant than glutamate 10111 (arising from oxaloacetate 1101 and [1,2-^13^C]acetyl-CoA) (Fig. 2G). These findings emphasize that positional ^13^C assignment detects the consequences of multiple routes of ^13^C entry into the TCA cycle, even among isotopomers with the same number of ^13^C nuclei.

### Limits of detection

We established the detection limits for this method using ^13^C-labeled standards, cell lines cultured with [U-^13^C]glucose, and tissues from mice infused with [U-^13^C]glutamine or [U-^13^C]glucose. We prepared two groups of standards. One set contained [1,2-^13^C]glutamate and [1,4-^13^C]aspartate at fractional enrichments of 1–5%. The second contained [3,4-^13^C]glutamate at fractional enrichments of 1–5%. The true fractions of each isotopomer are equal to the corresponding isotopologues, which were measured using a high resolution full scan on an orbitrap mass spectrometer. For the measured isotopomers, the relative errors were below 10% for isotopomers with at least 2% fractional enrichment and decreased further as the fractional enrichment increased (Fig, S2E). In isotopomers with 1– 2% enrichment, relative errors could be as high as 30% (Fig. S2E.) We emphasize that isotopomers at such low abundance would be difficult to detect at all using NMR, so even with these relative errors the new isotopomer method provides otherwise unavailable information.

We then determined the isotopomer distribution from two cell lines, Huh7 hepatoma cells and SFxL glioma cells, using roughly 1 million cells per sample. We performed serial dilutions until we obtained samples equivalent to 2,000 cells. Because these measurements are a mixture of isotopomers and their respective ratios, it is difficult to define the lowest limit for each individual isotopomer. To establish the minimum amount of material required, we sought to have relative standard deviation (RSD) less than 10% for fractional enrichments above 2%, and RSD less than 30% for fractional enrichments of 1–2%. We find that the isotopomer distributions are consistent down to approximately 16,000 cells (Fig. S3A-D).

We next took kidney, liver and brain tissues from healthy 12–16 week old NSG mice infused with [U-^13^C]glutamine. We prepared extracts from these tissues so that each injection into the LC-MS/MS would be equivalent to 1–2 mg of tissue. We then performed serial dilutions of the extracts until the injection amount was equivalent to 10 µg tissue samples. For isotopomers with enrichments of 1% or more, the distributions are consistent down to injections equivalent to 500 µg of tissue (Fig S3E,F). Similar to the experiments involving cultured cells, we used RSD cutoffs of less than 10% for fractional enrichments above 2%, and RSD less than 30% for fractional enrichments of 1–2%.

We then infused mice at different rates to examine isotopomer labeling at different levels of precursor labeling. Healthy 12–16 week old NSG mice were infused with [U-^13^C]glucose at one of four different rates. This produced a range of glucose m+6 enrichments in the brain at the end of the infusion, and labeling in both glutamate and aspartate correlated with glucose m+6 enrichment (Fig. S3G). Individual isotopomers of glutamate and aspartate occurred at varying levels, with glutamate 00011 being the most abundant across the range of glucose m+6 enrichments (Fig. S3G). Other isotopomers were also detected and became more prominent at higher glucose m+6 enrichments. This experiment demonstrates that several isotopomers can be detected from the mouse brain, even at tissue glucose enrichments under 5%.

### Detection of distinct modes of anaplerosis in cancer cell lines and mouse tissues.

**Figure 3.**
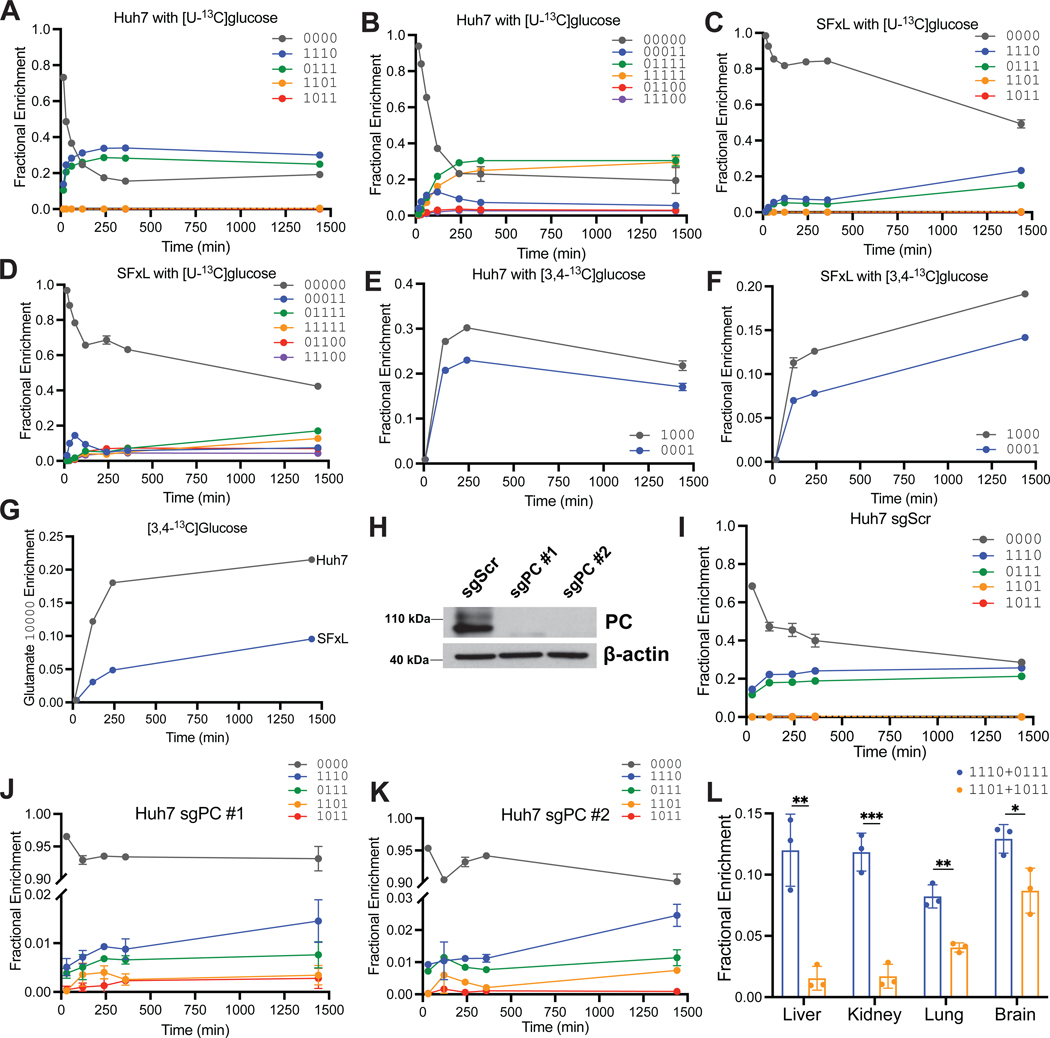
Kinetic analysis of glutamate isotopomers in cancer cells. (A-B) Time course of selected aspartate and glutamate isotopomers in Huh7 hepatoma cells cultured in medium containing [U-^13^C]glucose (3 biological replicates). (C-D) Time course of selected aspartate glutamate isotopomers in SFxL gliomas cells cultured in medium containing [U-^13^C]glucose (3 biological replicates). (E-G) Time course of selected aspartate and glutamate isotopomers in Huh7 hepatoma cells and SFxL gliomas cells cultured in medium containing [3,4-^13^C]glucose. In panels A-G, enrichment in glutamate and aspartate was assumed to be 0.0 at time 0 (3 biological replicates). (H) Western blot of PC in Huh7 hepatoma cells expressing control guide RNAs (sgScr) or guide RNAs targeting PC (sgPC #1, #2). (I-K) Time course of selected aspartate isotopomers in sgScr and sgPC Huh7 hepatoma cells cultured in medium containing [U-^13^C]glucose (3 biological replicates). (L) Selected aspartate M+3 isotopomers in mouse organs after infusion with [U-^13^C]glucose (3 biological replicates). P values: ns = P > 0.05; * =P ≤ 0.05; ** = P ≤ 0.01; *** = P ≤ 0.001; **** = P ≤ 0.0001. Unpaired t tests were used and the data are shown as mean ± standard deviation

Cancer cells in culture have substantial anaplerotic fluxes as described above for H460 cells. Glutamine catabolism and PC are two well-characterized modes of anaplerosis^23,24^. We previously analyzed ^13^C enrichment using NMR and non-positional MS to characterize anaplerosis in SFxL (high glutamine catabolism) and Huh7 (high PC) cells^24^. To test whether the LC-MS/MS method detects these differences, we cultured both cell lines with [U-^13^C]glucose and analyzed positional labeling with natural abundance correction. Over several hours, Huh7 cells displayed enhanced fractional accumulation of PC-dependent isotopomers of aspartate (1110 and 0111) and glutamate (11100 and 01100) comparing to SFxL cells (Fig. 3A-D). Huh7 cells also rapidly accumulated higher-ordered labeling in glutamate (01111 and 11111), in which the former arose from OAA 1110 and [1,2-^13^C]acetyl-CoA, and the latter from OAA 0111 and [1,2-^13^C]acetyl-CoA (Fig. 3A, B).

To further examine PC-dependent labeling, we used [3,4-^13^C]glucose. With this tracer, ^13^C enters the TCA cycle only via OAA as a consequence of PC activity (Fig. S4). In Huh7 cells, labeled aspartate appeared rapidly as 1000 and 0001, whereas these isotopomers accumulated slowly in SFxL cells (Fig. 3E,F). Glutamate 10000, which also arises downstream of PC, appeared more rapidly in Huh7 cells (Fig. 3G).

We next examined the effects of PC loss by depleting PC in Huh7 cells using CRISPR-Cas9 (Fig. 3H). During culture with [U-^13^C]glucose, control cells expressing a non-targeting guide RNA (sgScr) demonstrated rapid production of aspartate 1110 and 0111, similar to parental Huh7 cells (Fig. 3I). However, in the two PC-deficient lines, labeled forms of aspartate were reduced 10-fold or more (Fig. 3J,K).

Finally, we explored PC activity in mouse organs after infusing with [U-^13^C]glucose. In the liver and kidney, the sum of aspartate 1110 plus 0111 far exceeded the sum of aspartate 1101 plus 1011, indicating PC activity (Fig. 3L). A smaller but still positive difference was observed in lung and brain. These data are consistent with the known robust PC activity in liver and kidney.

### Application of LC-MS/MS to interpret labeling patterns in a mouse model of tumor growth.

**Figure 4.**
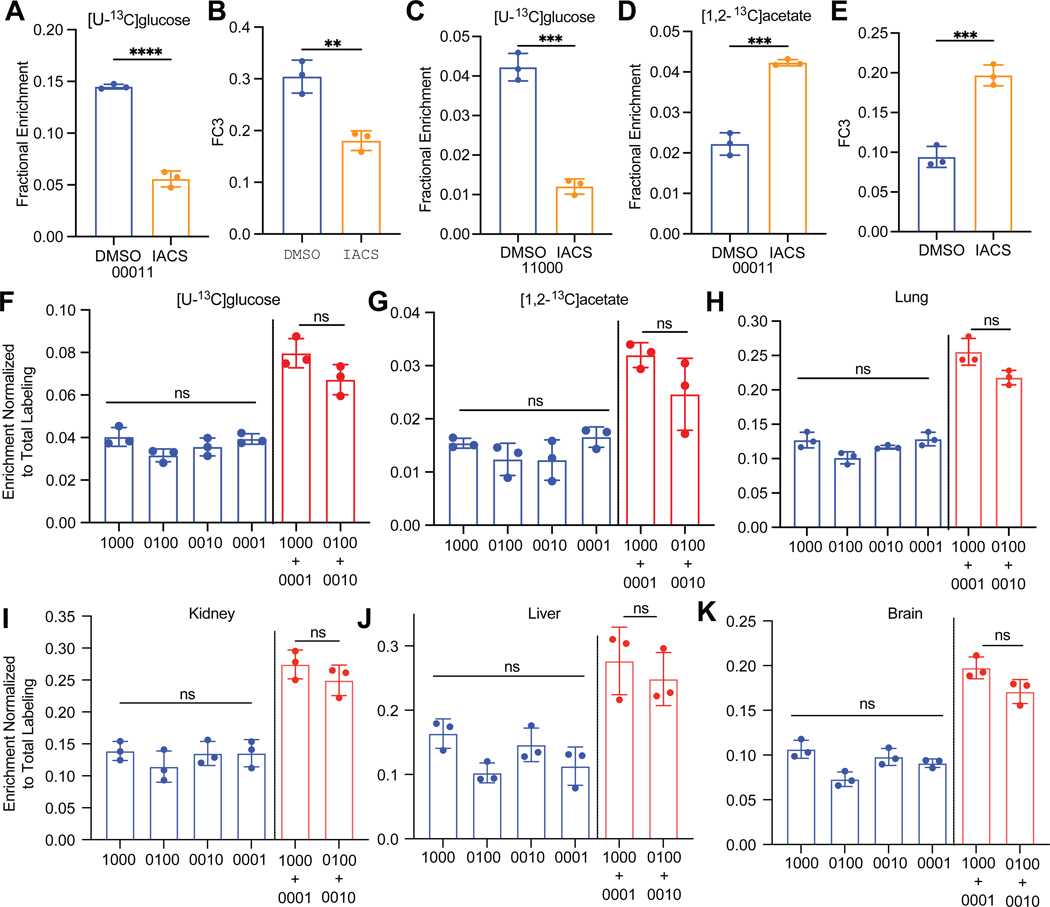
In vivo applications of LC-MS/MS isotopomer analysis. (A-C) Relative [4,5-^13^C]glutamate abundance (A), F_C3_ fraction (B) and abundance of [1,2^13^-C]glutamate as a fraction of the total glutamate pool in neuroblastoma xenografts after infusion with [U-^13^C]glucose and treatment with DMSO or IACS-010759 (3 biological replicates). (D-E) Relative [4,5-^13^C]glutamate abundance (D) and F_C3_ fraction (E) in neuroblastoma xenografts after infusion with [1.2-^13^C]acetate and treatment with DMSO or IACS-010759 (3 biological replicates). (F-G) Aspartate isotopomers from DMSO-treated neuroblastoma xenografts after infusion with [U-^13^C]glucose (F) or [1,2-^13^C]acetate (G) (3 biological replicates). (H-K) Aspartate M+1 isotopomers (normalized to total labeling) in mouse organs after infusion with [U-^13^C]glucose (3 biological replicates). P values: ns = P > 0.05; * =P ≤ 0.05; ** = P ≤ 0.01; *** = P ≤ 0.001; **** = P ≤ 0.0001. Unpaired t tests were used in panels A-E and the data are shown as mean ± standard deviation. An ordinary one way ANOVA was used for the comparison of m+1 isotopomers (blue) in panels F-K, while unpaired t tests were used for the comparison of labeling in outer m+1 isotopomers vs inner m+1 isotopomers (red). Data are shown as mean ± standard deviation.

We next used LC-MS/MS isotopomer analysis to assess labeling in tumors from mice infused with ^13^C-labeled nutrients. We generated SK-N-AS neuroblastoma xenografts, infused the mice with either [U-^13^C]glucose or [1,2-^13^C]acetate, and treated them with a vehicle control (DMSO) or IACS-010759, an inhibitor of Complex I of the electron transport chain (ETC). Because Complex I recycles NADH to NAD+, and PDH requires NAD+ as a cofactor, IACS-010759 reduces the fractional abundance of isotopomers arising from PDH^25^. Indeed, glutamate 00011 and F_C3_ were suppressed by IACS-010759 during infusion with [U-^13^C]glucose (Fig. 4A,B). Labeling in the second turn of the TCA cycle (e.g. glutamate 11000) was also suppressed by IACS-010759 (Fig. 4C). IACS-010759 increased rather than decreased glutamate 00011 and F_C3_ after infusion with [1,2-^13^C]acetate, which does not require PDH to label Ac-CoA (Fig. 4D,E). Therefore, the method detects the anticipated effects of Complex I inhibition in tumor-bearing mice.

A puzzling aspect of in vivo tumor metabolism studies is the presence of large M+1 isotopologue fractions in TCA cycle intermediates after infusion with tracers that produce [1,2-^13^C]acetyl-CoA (e.g. [U-^13^C]glucose)^26,27^. Explanations for these M+1 isotopologues include carboxylation of unlabeled intermediates using ^13^CO_2_ liberated during the oxidation of labeled fuels, and the progressive oxidation of M+2 isotopologues through multiple rounds of the TCA cycle^27,28^. Simple isotopologue analysis does not discriminate between these possibilities. However, the position of ^13^C within TCA cycle metabolites reflects the mechanism of label delivery to the cycle (Fig. S5A,B). Carboxylation reactions label the outer carbons of the product metabolite, and the label is subsequently lost as the product is oxidized. Labeling of citrate through condensation of [1,2-^13^C]acetyl-CoA and unlabeled OAA produces M+2 intermediates through the first turn of the cycle, with the ^13^C located at positions 4 and 5 of glutamate and then at either 1 and 2 or 3 and 4 of aspartate. With each subsequent turn, the likelihood of incorporating [1,2-^13^C]acetyl-CoA is proportional to its fractional enrichment, which is low in SK-N-AS tumors (F_C3_=0.3, Fig. 4B). Oxidation of intermediates labeled as M+2 on the first turn results in M+1 labeling, starting in turn 2, but the position of ^13^C is mixed between inner and outer carbons. Specifically, labeling in aspartate under this scenario is predicted to be distributed equally between inner and outer carbons, even with no contribution of ^13^CO_2_ recycling. Examination of M+1 aspartate labeling after natural abundance correction in SK-N-AS xenografts infused with [U-^13^C]glucose revealed nearly equal labeling of outer and inner carbons, with only a non-significant predominance of outer carbon labeling (Fig. 4F). Similar results were observed during infusions with [1,2-^13^C]acetate, although the overall labeling was lower (Fig. 4G). We also examined aspartate isotopomers in healthy mouse tissues after [U-^13^C]glucose infusion, and again did not detect an excess of outer carbon labeling (Fig. 4H-K). This suggests that TCA cycling rather than carboxylation is the major source of M+1 labeling in aspartate under these conditions.

### Application of LC-MS/MS isotopomer analysis to human cancer.

**Figure 5.**
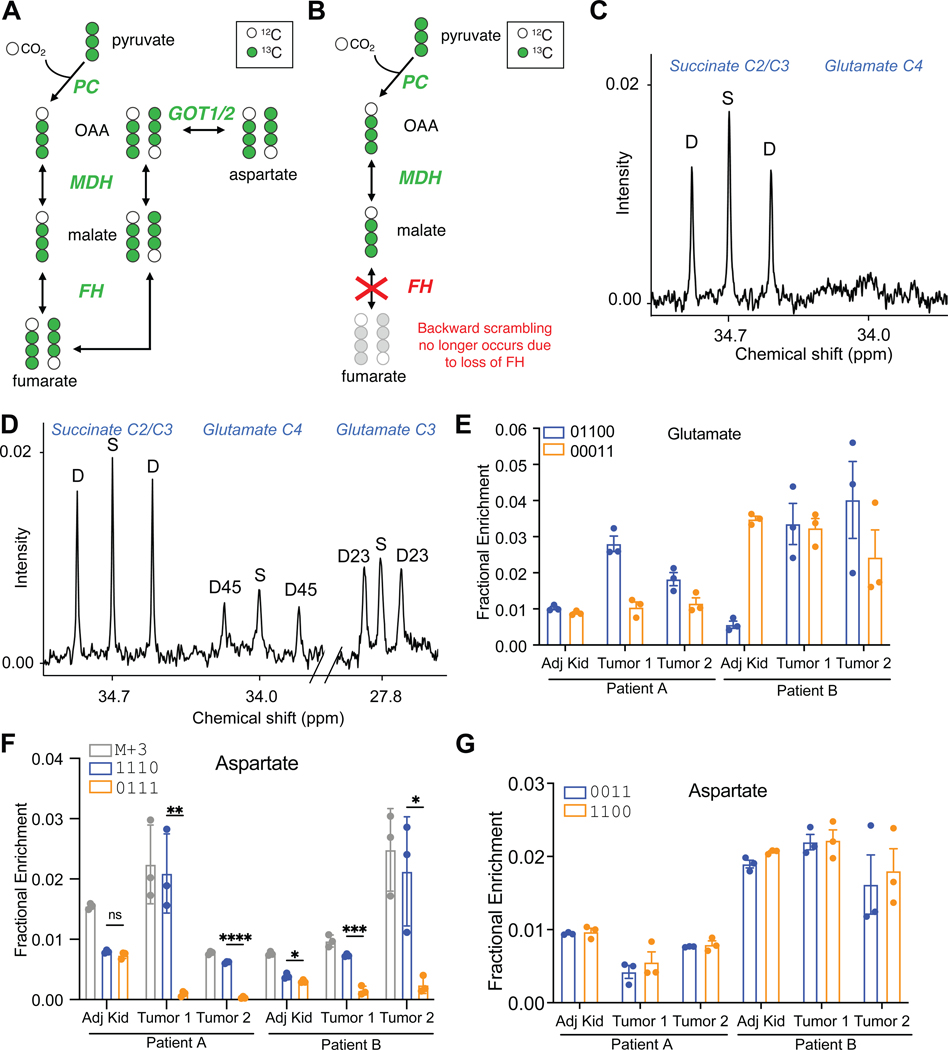
Isotopomer analysis from human kidney tumors. (A) Schematic of symmetric isotopomers of malate, fumarate, and aspartate arising from equilibration of [1,2,3-^13^C]oxaloacetate with malate and fumarate via malate dehydrogenase (MDH) and fumarate hydratase (FH). The activity of glutamic-oxaloacetatic transaminases (GOT1/2) ultimately produces equivalent abundances of [1,2,3-^13^C]aspartate and [2,3,4-^13^C]aspartate. (B) When FH is defective, equilibration of symmetric isotopomers does not occur, resulting in an excess of [1,2,3-^13^C]aspartate relative to [2,3,4-^13^C]aspartate (i.e. more aspartate 1110 than 0111). (C-D) Expanded ^13^C NMR spectra of glutamate C4 and C3 from two fragments of a human FHdRCC after infusion with [U-^13^C]glucose during nephrectomy. (E) Selected glutamate isotopomers analyzed by LC-MS/MS from fragments of tumor and adjacent, non-malignant kidney (Adj Kid) tissue obtained after infusion with [U-^13^C]glucose during nephrectomy in two patients (3 biological replicates). (F-G) Relative contributions of selected aspartate M+3 (F) and M+2 (G) isotopomers in these tissue fragments (3 biological replicates). P values: ns = P > 0.05; * =P ≤ 0.05; ** = P ≤ 0.01; *** = P ≤ 0.001; **** = P ≤ 0.0001. An unpaired t test was used in panel F.

In ^13^C infusions in cancer patients, at least 100 mg of tissue is used to assess ^13^C labeling by NMR^11,27,29,30^. The practicalities of ^13^C stable isotope infusions in patients have been covered elsewhere^31^. In tumors from the brain and lung, this approach provides adequate signal to noise ratios (SNR) to observe some isotopomers of glutamate and aspartate. However, using samples this large obscures the regional metabolic heterogeneity characteristic of solid tumors in humans^12,13,27^. The problem is compounded in tumors from the kidney, which are both highly heterogeneous and characterized by low labeling of TCA cycle intermediates from [U-^13^C]glucose^29^. We compared NMR and LC-MS/MS analysis of tumors from two kidney cancer patients with FH deficient renal cell carcinoma (FHdRCC). Both patients were infused with [U-^13^C]glucose during nephrectomy. FHdRCC tumors lack fumarate hydratase (FH) and are therefore expected to have suppressed TCA cycle function (Fig. 5A,B). After nephrectomy, samples were obtained from the nonmalignant kidney and from two tumor regions. In one sample from patient A’s tumor, glutamate C4 labeling could not be detected by NMR (Fig. 5C). In the other sample, weighing 460 mg, NMR detected ^13^C multiplets in glutamate C3 and C4, but the SNR was low (Fig. 5D). Multiplets at succinate C2/3 were prominent in both samples, perhaps reflecting succinate accumulation resulting from FH loss.

For LC-MS/MS, we used three small (10–20 mg) fragments of each sample. Of the observed ion pairs monitored by MRM in these samples, 60–100% had SNRs over 10 (Fig. S6A,B). Natural abundance corrected glutamate labeling had hallmarks of both PDH and PC activity, with 2–4% of the pool labeled as glutamate 01100 and 1–3% labeled as glutamate 00011 (Fig. 5E). Because analysis of glutamate 01100 is complicated by potential calculation error, we also examined glutamate 11100 proceeding from PC-dependent production of aspartate 0111. However, the abundance of this isotopomer was negligible. Interestingly, aspartate 1110 exceeds aspartate 0111 in both fragments of the tumor, and aspartate 1110 accounts for most of the m+3 isotopologue (Fig. 5F, Fig. S6C-F). These patterns imply activity of PC to label OAA/aspartate, but poor equilibration with the fumarate pool, as predicted by loss of FH activity within these tumors (Fig. 5A,B). In the nonmalignant kidney fragments from both patients, where FH activity persists, aspartate 1110 and 0111 are similar (Fig. 5F, Fig. S6C-F), indicating that the lack of symmetrization in the tumors was not an artifact of tissue handling during the surgery. It is also interesting that aspartate isotopomers arising from PDH (1100 and 0011) were equivalent to each other in both the tumors and the kidney samples (Fig. 5G), indicating that the expected symmetry is achieved in isotopomers arising from progression of the cycle from citrate to OAA/aspartate. It is unclear whether the equivalent levels of aspartate 1100 and 0011 in the tumors reflects a small amount of residual FH activity in tumor cells, or whether part of the aspartate pool in these samples was taken up from other tissues with functional FH. Altogether, this analysis reveals the ability of the LC-MS/MS method to detect regional metabolic heterogeneity from small samples of human tumors, to provide information about routes of carbon entry into and processing by the TCA cycle, and more generally why reporting ^13^C position complements isotopologue analysis in metabolites related to the TCA cycle.

## DISCUSSION

Although ^13^C tracers are excellent tools to assess metabolic activity, simple isotopologue analysis (by far the most common mass spectrometry technique currently used in such experiments) reports only a fraction of the information encoded by ^13^C labeling ^1^. We describe a method that combines the high sensitivity of mass spectrometry with the positional information usually determined by NMR. A primary objective was to be able to report the isotopomers of glutamate required in calculations of metabolic parameters relevant to the TCA cycle, including anaplerosis and enrichment of the acetyl-CoA pool that supplies citrate synthesis. In addition to this subset of glutamate isotopomers, the method provides direct or indirect information about the complete set of isotopomers from both glutamate and aspartate. The complementary information provided by all these labeled species provides a highly detailed analysis of the TCA cycle, while requiring far less material than what is needed for NMR.

We believe this approach will have particular value in human and other in vivo studies when performing multiple tracer experiments is impractical or impossible. For example, specialized tracers can be used to improve certainty about specific reactions in the metabolic network. While [3,4-^13^C]glucose can be used to probe PC activity, the high cost and otherwise low information yield of this tracer makes it less appealing than [U-^13^C]glucose in human studies. The isotopomer method reported here increases clarity about PC activity from [U-^13^C]glucose tracing data, and is therefore a considerable advantage. The high sensitivity of the method is also an obvious advantage for human cancer studies where metabolite labeling tends to be low due to the sub-maximal enrichment of the circulating precursor pool. Although it is possible to obtain informative 1D ^13^C NMR spectra from tumors, the large samples required make it difficult to study regional heterogeneity of ^13^C signatures, which can be substantial in solid tumors^12,13,27^. The larger the sample needed for analysis, the more averaging of heterogeneous metabolic features must occur. Therefore, using larger samples can obscure subtle metabolic features and make it difficult to study how these features are regulated by regional differences in histology, gene expression, proximity to vasculature, et cetera.

While ^1^H detection of ^13^C isotopomer distributions is feasible and much more sensitive than 1D ^13^C NMR, this technique is limited to the analysis of protonated carbons. The carboxyl carbons are also important indicators of metabolism, as illustrated for aspartate where the external carboxyl carbons and internal protonated carbons may originate from different sources. To quantitatively evaluate multiplets at the carboxyl positions of glutamate and aspartate, 1D ^13^C spectroscopy was the most appropriate alternative. The high sensitivity of the method described here should make it possible to perform isotopomer analysis in very small tumor samples. Combining this method with MS imaging is a particularly exciting application, but sensitivity would need to be further improved.

Finally, a challenge of in vivo isotope tracing studies in cancer is the appearance of unexpected isotopologue distributions. Verifying the origin of such labeling patterns is straightforward in cultured cells, where silencing enzymes of interest makes it possible to identify the responsible pathway. This is more difficult in mice and impossible in patients. The prominent M+1 labeling of TCA cycle intermediates after infusions with uniformly ^13^C-labeled nutrients in vivo is an example of this challenge. While M+1 fractions tend to be small in cell culture, this fraction appears quickly in vivo and matches or exceeds the more familiar M+2 fraction. Positional specificity is informative about the origins of the label. If ^13^CO_2_ liberated from labeled substrates is used as substrate in carboxylation reactions, it is predicted to reside on the outer positions (i.e. carbons 1 and 4) of 4-carbon intermediates like aspartate. These labels are largely lost as ^13^CO_2_ during subsequent decarboxylation reactions rather than transferred to internal carbons. On the other hand, PDH followed by successive turns of the cycle produces labels distributed evenly between internal and external carbons. Simulations of TCA cycle activity indicate that when F_C3_ is low, these successive rounds of the cycle can produce high levels of M+1 isotopologues, sometimes exceeding the M+2 fraction, even in the absence of ^13^CO_2_ fixation^27^. We detect nearly identical labeling of internal and external aspartate carbons after infusion with [U-^13^C]glucose or [1,2-^13^C]acetate. This suggests that most of the M+1 labeling in TCA cycle intermediates from these tracers arises downstream of PDH rather than carboxylation and ^13^CO_2_ recycling. We emphasize that the data do not rule out some contribution of ^13^CO_2_ recycling, but the simplest interpretation is that this is a minor component of the M+1 labeling observed in vivo, at least from [U-^13^C]glucose and [1,2-^13^C]acetate.

In summary, we established an LC-MS/MS method to identify complete isotopomer fractions of glutamate or aspartate. The isotopomer information acquired using this method can detect PDH activity, PC activity, anaplerosis and somatic loss of FH in tumors. These examples demonstrate the utility of this approach in tumor metabolism and other biological systems.

### Limitations of Study

Although the information yield from this method is greater than NMR, the technique is analytically demanding. For straightforward applications (e.g. calculation of F_C3_) when the SNR is high and tissue is abundant, NMR may still be preferable. The presented MRM method is also a low resolution MS method that does not distinguish between ^15^N and ^13^C. Since multiple fragments contain nitrogen, the data needs to be corrected for natural abundance isotopes using the provided matrix. Transferring the presented method to a high resolution mass spectrometry instrument may solve the error presented by natural abundance of other elements. Although the approach provides information about all isotopomers of glutamate and aspartate, it results in substantial relative errors for some isotopomers, particularly when the fractional enrichment is low. The impact of these errors can be reduced somewhat by choosing tracers that limit the production of mixed acetyl-CoA isotopomers, and by incorporating complementary information (e.g. F_C3_) to reduce uncertainty about problematic isotopomers.

## STAR METHODS 

### Lead Contact

Further information and requests for resources and reagents should be directed to the lead contact, Ralph DeBerardinis, MD, PhD. Email: Ralph.DeBerardinis@utsouthwestern.edu

### Materials Availability

PC knockout cell lines are available upon request.

### Data and Code Availability

The script for natural isotope abundance correction matrix, non-negative least square regression, and error estimation have been deposited at the Github repository (https://github.com/RJDlab/Glu_Asp_Isotopomers). All source data is provided in Data S1. All raw data is provided in Supplemental Data III.

#### Experimental Model and Study Participants Details

All mouse infusions were performed in compliance with protocols approved by the Institutional Animal Care and Use Committee at the University of Texas Southwestern Medical Center (Protocol 2016–101694). NOD.CB17-Prkdc^*scid*^Il2rg^tm1Wjl^/SzJ (NSG) male and female mice aged 4–8 weeks old were purchased from the Jackson Laboratory. All mice were housed in the Animal Resource Center at the University of Texas Southwestern Medical Center under a 12 hr light-dark cycle and fed ad libitum.

Human subjects research was approved by the Institutional Review Board of UT Southwestern Medical Center. After obtaining informed consent, patients were enrolled on protocol STU062010–157 (Clinicaltrials.gov protocol NCT01888082): An Investigation of Tumor Metabolism in Patients Undergoing Surgical Resection. Patients were selected for inclusion based on imaging and clinical features consistent with renal cell carcinoma.

### Chemicals

All chemicals and reagents were LC-MS grade or higher. Sterile, pyrogen free [U-^13^C]glucose was purchased from Cambridge Isotope Laboratories for human infusions (CLM-1396-MPT-PK). For mouse infusions, [1,2-^13^C]acetate was purchased from Sigma-Aldrich (663859) and [U-^13^C]glucose was purchased from Cambridge Isotope Laboratories (CLM-1396).

### Human Infusions

Sterile, pyrogen free [U-^13^C]glucose was infused at the time of nephrectomy. A peripheral intravenous line was placed on the morning of surgery and labeled glucose was infused as a bolus of 8g over 10 minutes followed by 8g per hour as a continuous infusion. Standard procedures were followed for tumor resection and tissue fragments were flash frozen in liquid nitrogen. All diagnoses were made by the attending clinical pathologist. These infusion parameters are consistent with previously published work (Faubert et al 2021, Courtney et al 2018, Faubert et al., 2017; Hensley et al., 2016; Maher et al., 2012; Mashimo et al., 2014).

### Mouse Infusions

For tumor studies, subcutaneous injections were done in the right flank of NOD.CB17-Prkdc^*scid*^Il2rg^tm1Wjl^/SzJ (NSG) male and female mice aged 4–8 weeks old. To establish xenografts from cancer cell lines, suspensions of neuroblastoma cells were prepared for injection in RPMI 1640 media with Matrigel (CB-40234; Fisher Scientific). 50μL of the cell suspension was combined with 50μL Matrigel for a total volume of 100μL per mouse. For studies that involved treatment with the complex I inhibitor (IACS-010759, ChemieTek), the mice were administered IACS-010759 by oral gavage every day for 5 days (10 mg/kg body mass in 100 μL of 0.5% methylcellulose and 4% DMSO) once subcutaneous tumors reached 200 mm^3^. On the 5^th^ day, mice were infused with [U-^13^C]glucose or [1,2-^13^C]acetate and tumors were harvested 3–5 hours following the last treatment dose, depending on the infusion. Mice were weighed and subcutaneous tumors measured at the beginning and end of the 5-day treatment period. Mice were anesthetized and then a 27-gauge catheter was placed in the lateral tail vein under anesthesia. [1,2-^13^C]acetate (Sigma-Aldrich, 663859) was delivered as a bolus of 0.3 mg/g body mass over 1 min in 150 μL of saline, followed by continuous infusion of 0.0069 mg/g body mass/min for 3 hours in a volume of 150 μL/hour. [U-^13^C]glucose (Cambridge Isotope Laboratories, CLM-1396) was intravenously infused as a bolus of 0.4125 mg/g body mass over 1 min in 125 μL of saline, followed by continuous infusion of 0.008 mg/g body mass/min for 3 hours in a volume of 150 μL/hour. For [U-^13^C]glutamine (Cambridge Isotope Laboratories, CLM-1822) infusions, the total delivered dose was 1.73 g/kg dissolved in 1.5 mL saline. The glutamine solution was administered at a bolus rate of 150 μL/min for 1 min followed by a continuous infusion rate of 2.5 μL/min for 5 hours. At the end of the infusion, mice were euthanized and tumors and/or organs were harvested and immediately frozen in liquid nitrogen. For healthy, non-tumor bearing mouse infusions with [U-^13^C]glucose shown in Figure S3G, [U-^13^C]glucose (Cambridge Isotope Laboratories, CLM-1396) was intravenously infused for 180 minutes at continuous rates of either 3.3 mg [U-^13^C]glucose/kg mouse body weight/min, 1.65 mg/kg/min, 0.83 mg/kg/min, or 0.41 mg/kg/min with no bolus dose. To assess the fractional enrichments in plasma, roughly 20 μL of blood was obtained after 30, 60, 120, and 180 minutes of infusion from retro-orbital bleeds.

### Cell lines and culture conditions

H460 cells were obtained from the Hamon Cancer Center Collection (University of Texas Southwestern Medical Center) and maintained in RPMI-1640 supplemented with penicillin–streptomycin and 5% fetal bovine serum (FBS) at 37°C in a humidified atmosphere containing 5% CO_2_. SF188-derived glioblastoma cells overexpressing human Bcl-xL (SFxL) were previously reported^32^ and Huh7 hepatocellular carcinoma cells were a gift from Professors Michael S. Brown and Joseph L. Goldstein. SFxL and Huh-7 were maintained in Dulbecco’s modified Eagle medium (DMEM) supplemented with penicillin–streptomycin and 10% FBS at 37°C in a humidified atmosphere containing 5% CO_2_. All cell lines were validated by DNA fingerprinting using the PowerPlex 1.2 kit (Promega) and were confirmed to be mycoplasma-free using the e-Myco kit (Boca Scientific).

### PC depletion in Huh7 cells

lentiCRISPR v2 was a gift from Feng Zhang (Addgene plasmid # 52961; http://n2t.net/addgene:52961; RRID:Addgene_52961). Guide RNA oligos were ordered from IDT Company. Guide RNAs (sgScr: 5’ to 3’ TTCTTAGAAGTTGCTCCACG, sgPC#1: 5’ to 3’ CAGGCCGCGGCCGATGAGAT, sgPC#2: 5’ to 3’ ACAGGTGTTCCCGTTGTCCC) were cloned into LentiCRISPRv2 ^33^, then transfected into 293T cells using Lipofectamine 3000 (Invitrogen, L3000001) with a 2:1 ratio of psPAX2: pMD2G. Medium containing viral particles was collected and filtered using 0.45µM membrane filters 48 hours after the transfection. Huh7 cells were cultured in media containing lentivirus and 4 µg/mL polybrene (Sigma, TR-1003-G) for 24 hours followed by selection in 5 µg/mL puromycin until the uninfected control cells died.

### Western Blots

Cells were lysed in RIPA buffer (Boston BioProducts, BP-115) containing protease and phosphatase inhibitors (Thermo Fisher Sciences, 78444), then centrifuged at 4°C for 10 minutes at ~20,160 g. Supernatants were transferred to new pre-chilled 1.5 mL tubes and protein concentrations were quantified using the Thermo Fisher Pierce BCA Assay Kit (Thermo Fisher, 23225). Protein lysates were resolved via SDS-PAGE and transferred to PVDF membranes. Membranes was blocked in 5% bovine serum albumin (BSA) in Tris Buffered Saline with Tween-20 (TBST (20 mM Tris pH 7.5, 150 mM NaCl, 0.1% Tween-20)) and then incubated with primary antibodies (PC: Proteintech, 16588–1-AP, 1:1000 dilution, or β-actin: Cell Signaling, 8457S, 1:1000 dilution) in TBST supplemented with 5% BSA at 4°C overnight. Primary antibodies were detected with a horseradish peroxidase-conjugated secondary antibody (Cell Signaling Technology, 7074S, 1:2000 dilution) for 1 hour followed by exposure to ECL reagents (Fisher Scientific, PI32106).

### Standard Preparation for LC-MS/MS

Standards were prepared as aqueous stock solutions of L-glutamate (1 mg/mL), [1,2-^13^C]L-glutamate (1 mg/mL), [3,4-^13^C]L-glutamate (1 mg/mL), L-aspartate (1 mg/mL), and [1,4-^13^C]L-aspartate (1 mg/mL).

The Group A solution contained [1,2-^13^C]glutamate and [1,4-^13^C]L-aspartate at fractional enrichments of 1%, 2%, 3%, 4%, or 5%. The solution was prepared by mixing [1,2-^13^C]L-glutamate and [1,4-^13^C]L-aspartate with the L-glutamate and L-aspartate stock solutions, respectively. The Group B solution contained [3,4-^13^C]L-glutamate at fractional enrichments of 1%, 2%, 3%, 4%, or 5% and was prepared by mixing the [3,4-^13^C]L-glutamate solution with the L-glutamate stock solution. The groups A and B solutions were then combined and diluted 10,000 times or 20,000 times with 80% acetonitrile to prepare standards for error analysis.

### Sample preparation for LC-MS/MS

Tissue samples (2–5mg) were cut on a surface cooled with dry ice, transferred into a 1 mL microcentrifuge tube on dry ice, and ground with a pellet pestle. Ice cold 80% (vol/vol) acetonitrile in water (200 µL) was added to the tube, and the tissue was homogenized until no visible chunks remained.

Cells were plated at 1 × 10^5^ cells per 6-cm plate 16 h before labeling. The next day, cells were incubated in glucose/glutamine-free DMEM containing 20 mM [U-^13^C]glucose or [3,4-^13^C]glucose. At the desired time, the medium was aspirated and cells were rinsed with ice cold saline. The saline was aspirated, 80% (vol/vol) acetonitrile in water was added and the cells were collected with a cell scraper. The resulting mixture was transferred to a microcentrifuge tube and subjected to three freeze-thaw cycles in liquid nitrogen and a 37^o^C water bath. The samples were vortexed for 1 min before centrifugation at ~20,160 g for 15 min at 4°C. The metabolite-containing supernatant (195 µL) was transferred into pre-cut Bond^®^ Elut PH (100 mg, 1 mL) SPE cartridges (Agilent, 12102005) placed in 1.7 mL microcentrifuge tubes and then centrifuged at ~3000 g for 3 min. Alternatively, the supernatants were passed through Oasis® HLB LP 96-well plate 60µm (60mg) SPE cartridges (Waters, 186000679). The filtrate was then transferred to LC/MS vials for analysis.

### LC-MS/MS analysis

Samples were analyzed on an AB Sciex 6500 QTRAP liquid chromatography/mass spectrometer (Applied Biosystems SCIEX) equipped with a vacuum degasser, quaternary pump, autosampler, thermostatted column compartment and triple quadrupole/ion trap mass spectrometer with electrospray ionization interface, and controlled by AB Sciex Analyst 1.6.1 Software. SeQuant® ZIC®-pHILIC 5µm polymer (150mm×2.1mm) columns were used for separation. Solvents for the mobile phase were 10 mM ammonium acetate aqueous (pH 9.8 adjusted with ammonium hydroxide (A) and pure acetonitrile (B). The gradient elution was: 0–20 min, linear gradient 90–65% B, 20–23 min, linear gradient 65–30% B, 23–28 min, 30% B, and 28–30 min, linear gradient 30–90% B then reconditioning the column with 90% B for 5 min. The flow-rate was 0.2 ml/min and the column was operated at 40°C.

### ^13^C Nuclear Magnetic Resonance Spectroscopy

For the cell culture experiments, H460 cells were plated at 5 × 10^6^ cells per 15 cm plate in three plates and allowed to adhere for 16 h before labelling. The next day, cells were incubated in glucose-free DMEM media containing 20 mM [U-^13^C]glucose for 24h before collection. The medium was aspirated and cells were rinsed with ice cold saline. The saline was aspirated and 80% (vol/vol) acetonitrile in water (5 mL) was added to the cells. The cells were scraped with a cell scraper. The mixtures were transferred into microcentrifuge tubes (0.5mL each) and subjected to three freeze-thaw cycles between liquid nitrogen and a 37°C water bath. The samples were vortexed for 1 min before centrifugation at ~20,160 g for 15 min at 4°C and the supernatants were centrifuged again at ~20,160 g for 15 min at 4°C before being dried at room temperature in a Speedvac system (Thermo Scientific, Waltham, MA).

The sample for ^13^C NMR analysis was prepared by dissolving the dried residue in 54 μL of 50 mM sodium phosphate buffer in D_2_O containing 2 mM ethylenediaminetetraacetic acid (EDTA). A 6 μL solution of 0.5 mM deuterated sodium 3-trimethylsilyl-1-propane sulphonate (d_6_-DSS) (internal standard) and 0.2% sodium azide (NaN_3_) in D_2_O was added to the sample. The solution was vortexed thoroughly, centrifuged at 10,000 g for 15 minutes and supernatant was loaded into a 1.5 mm NMR tube. ^13^C NMR data were acquired using a 14.1 T NMR magnet equipped with a ^13^C-optimized home-built high temperature superconducting (HTS) probe^34^ and VNMRJ Version-4.0 software. The following parameters were used to acquire ^13^C NMR data: number of scans = 30412, acquisition time (AQ) = 1.5s, relaxation delay (d1) = 1.5s, flip angle = 45°, acquired size = 54k. The ^13^C NMR spectrum was Fourier Transformed with an exponential line-broadening factor of 0.5 Hz, zero filling to 128k data points, and applying Whittaker smother baseline correction in MestReNova v14.0.1–23284 (Mestrelab Research S.L.). Mixed Gaussian/Lorentzian shape was used in the line fitting tool to fit the peak area of the multiplets for each of the ^13^C NMR peaks of glutamate and aspartate.

For NMR of samples from FHdRCC patients, frozen tissue samples (0.45 – 0.48 g) were powdered using a mortar and pestle chilled with liquid nitrogen. Ground tissue was transferred into a 15-mL conical tube containing perchloric acid solution (10%; 3 mL) on ice, vortexed for 1 min, and centrifuged at 25,000 g and 4 °C for 10 min. The supernatant was transferred to a new 15-mL conical tube, and the extraction was repeated by adding perchloric acid solution to the pellet. The combined supernatant was neutralized with KOH, centrifuged, and the supernatant was transferred to a 20-mL glass vial. After drying under vacuum, the extracts were dissolved in D_2_O (200 μL) containing 4,4-dimethyl-4-silapentane-1-sulfonic acid (5 mM), centrifuged at 20,000 g for 5 min and the supernatant was transferred to a 3-mm NMR tube. For samples from FHdRCC patients or H460 Cells, NMR spectra were collected using a 14.1 T spectrometer (Varian INOVA, Agilent, Santa Clara, CA) equipped with a 3-mm broadband probe with the observe coil tuned to ^13^C (150 MHz). NMR spectra were acquired using a 45° pulse, a 36,765-Hz sweep width, 55,147 data points, and a 1.5-sec acquisition time with 1.5-s interpulse delay at 25°C. Proton decoupling was performed using a standard WALTZ-16 pulse sequence. Spectra were averaged with 25,000 scans and a line broadening of 0.5 Hz was applied prior to Fourier transformation. Spectra were analyzed using ACD/Labs NMR spectral analysis program (Advanced Chemistry Development, Inc., Toronto, Canada).

### Nonnegative least square regression

Nonnegative least square regression was implemented using the glmnet R package^35^, with the following parameters: lambda = 0, lower.limits = 0, intercept = FALSE and thresh = 1e-30, such that the regression is not regularized, the coefficients are bounded between 0 and 1, and the convergence threshold is small but can generally be reached within the default number of iterations. We compared this method to other R packages with Lawson-Hanson’s algorithm^36^ implementation such as in the NNLS^37^ or pracma^38^ R packages and found the glmnet-based method to provide the lowest error from our simulation error estimation (Supplementary Data I Fig SD-I 1 and 2).

### Natural Abundance Correction

The following isotopes were considered during natural abundance correction: ^2^H (0.0156%), ^13^C (1.082%), ^15^N (0.366%), ^17^O (0.038%), and ^18^O (0.204%). All possible ^2^H, ^13^C, ^15^N, ^17^O, and ^18^O isotopomers of the precursor and product ions are listed and their probabilities are calculated and assigned to each MRM transition. The calculation matrices of glutamate and aspartate with or without natural isotope abundance correction are available in Supplemental Data II. The R script used for calculating the correction matrix can be found at the GitHub repository (https://github.com/RJDlab/Glu_Asp_Isotopomers).

## STATISTICAL ANALYSIS

Samples were analyzed as described in the figure legends. Data were considered significant if p<0.05. Statistics were calculated using PRISM software, and statistical details can be found in the figure legends for each figure.

## KEY RESOURCES TABLE

## Supplementary Material

1 Data S1. Unprocessed source data underlying all blots and graphs. Related to Figures 1–5 and Figures S2, S3, and S6

2 Supplemental Data I: MRM methods for glutamate and aspartate isotopomer analysis. Related to Figure 1 and STAR Methods.

3 Supplemental Data II: Calculation matrices of glutamate and aspartate. Related to STAR Methods.

4 Supplemental Data III: Raw data for corresponding figures. Related to Figures 1–5 and Figures S2, S3, and S6.

5

## Figures and Tables

**Table:** 

REAGENT or RESOURCE	SOURCE	IDENTIFIER
Antibodies		
Pyruvate Carboxylase Antibody	Proteintech	16588-1-AP
β-actin Antibody	Cell Signaling Technology	8457
Anti-rabbit IgG, HRP-linked Antibody	Cell Signaling Technology	7074
Bacterial and Virus Strains		
LentiCRISPR v2	Addgene	52961
pMD2.G	Addgene	12259
psPAX2	Addgene	12260
Biological Samples		
Matrigel	Fisher Scientific	CB-40234
Chemicals, Peptides, and Recombinant Proteins		
[U-^13^C]glucose	Cambridge Isotope Laboratories	CLM-1396
[U-^13^C]glutamine	Cambridge Isotope Laboratories	CLM-1822
[1,2-^13^C]acetate	Sigma Aldrich	663859
[3,4-^13^C]glucose	Cambridge Isotope Laboratories	CLM-6750
[1,4-^13^C]aspartate	Cambridge Isotope Laboratories	CLM-4455
[4-^13^C]aspartate	Sigma Aldrich	489999
[1,2-^13^C]glutamate	Cambridge Isotope Laboratories	CLM-2024
[3,4-^13^C]glutamate	Cambridge Isotope Laboratories	CLM-3646
L-aspartate	Sigma Aldrich	A9256
L-glutamine	Sigma Aldrich	G3126
IACS-010759	ChemieTek	CT-IACS107
(Hydroxypropyl)methyl cellulose	Sigma Aldrich	09963
Dimethyl sulfoxide (DMSO)	Sigma Aldrich	D1435
0.9% Sodium Chloride Solution	Baxter	N/A
RPMI-1640	Sigma Aldrich	R8758
Dulbecco′s Modified Eagle′s Medium (DMEM)	Sigma Aldrich	D5796
Dulbecco′s Modified Eagle′s Medium Powder, no glucose, glutamine, phenol red, sodium pyruvate and sodium bicarbonate	Sigma Aldrich	D503010
Polybrene	Sigma Aldrich	TR-1003-G
Lipofectamine^™^ 3000 Transfection Reagent	Invitrogen	L3000001
Puromycin	Fisher Scientific	NC9138068
RIPA Buffer	Boston BioProducts	BP-115
Halt^™^ Protease and Phosphatase Inhibitor Cocktail (100X)	Thermo Scientific	78444
Bovine Serum Albumin	Sigma Aldrich	A2153
Pierce^™^ ECL Western Blotting Substrate	Thermo Scientific	32106X4
Acetonitrile, Optima^™^ LC/MS Grade	Fisher Scientific	A955-4
Water, Optima^™^ LC/MS Grade	Fisher Scientific	W64
Ammonium acetate	Sigma Aldrich	431311
Methanol, Optima^™^ LC/MS Grade	Fisher Scientific	A456-4
Ammonium Hydroxide, Optima^™^	Fisher Scientific	A470-250
Formic Acid, 99.0+%, Optima^™^ LC/MS Grade	Fisher Scientific	A117-50
Potassium Phosphate Monobasic (Crystalline/Certified ACS)	Fisher Scientific	P285
D_2_O, 99.9%	Cambridge Isotope Laboratories	DLM-4
Ethylenediaminetetraacetic Acid (EDTA), Disodium Salt Dihydrate (Crystalline/Certified ACS)	Fisher Scientific	S311
Deuterated sodium 3-trimethylsilyl-1-propane sulfonic acid (d_6_-DSS)	Chenomx	IS-2
Sodium Azide, Crystalline	Fisher Scientific	S227I-25
Perchloric Acid, ACS, 60–62%	Fisher Scientific	AA33263AP
Potassium Hydroxide (Pellets/Certified ACS)	Fisher Scientific	P250
Sodium Hydroxide (Pellets/Certified ACS)	Fisher Scientific	S318
Hydrochloric Acid Solution, 6N (Certified)	Fisher Scientific	SA56
Critical Commercial Assays		
Pierce^™^ BCA Protein Assay Kit	Thermo Scientific	23225
e-Myco Mycoplasma PCR Detection Kit	Boca Scientific	25235
Experimental Models: Cell Lines		
Human: H460	Hamon Cancer Center Collection at the University of Texas Southwestern Medical Center	N/A
Human: Huh7	Gift from Michael S. Brown and Joseph L. Goldstein	N/A
Human: SK-N-AS	American Type Culture Collection (ATCC)	CRL-2137
Human: 293T	American Type Culture Collection (ATCC)	CRL-3216
Experimental Models: Organisms/Strains		
Mouse: NOD.CB17-Prkdc^*scid*^Il2rg^tm1Wjl^/SzJ (NSG)	The Jackson Laboratory	N/A
Oligonucleotides		
sgScramble	Integrated DNA Technologies (IDT)	TTCTTAGAAGTTGCTCCACG
sgPC #1	Integrated DNA Technologies (IDT)	CAGGCCGCGGCCGATGAGAT
sgPC #2	Integrated DNA Technologies (IDT)	ACAGGTGTTCCCGTTGTCCC
Deposited Data		
Analyzed data for all Figures	This paper	Data S1
Raw Data for all Figures	This paper	Supplemental Data III: Raw data for corresponding figures
Scripts for Natural isotope abundance correction matrix, Non-negative least square regression, and Error estimation	This paper	https://github.com/RJDlab/Glu_Asp_Isotopomers
Software and Algorithms		
Prism Graphpad	Graphpad Software	N/A
R Studio	Posit Software	N/A
NMR Spectrus Processor	ACD/Labs	N/A
AB Sciex Analyst 1.6.1 Software	Sciex	N/A
VNMRJ Version-4.0 software	Agilent	N/A
MestReNova v14.0.1–23284	Mestrelab Research S.L.	N/A
Other		
SeQuant^®^ ZIC^®^-pHILIC 5µm polymer 150 × 2.1 mm	Millipore Sigma	1504600001
Oasis HLB 96-well Plate, 60 mg Sorbent per Well, 60 µm	Fisher Scientific	50-818-654
14.1 T spectrometer	Varian INOVA, Agilent	N/A
QTRAP 6500	Sciex	N/A

## Highlights:

- MS-based isotopomer method requires less than 1% of sample required for NMR
- Isotopomer distributions provide detail about TCA cycle labeling from ^13^C tracers
- MS method is particularly useful in human and mouse isotope tracing experiments

## References

[1] Buescher JM, Antoniewicz MR, Boros LG, Burgess SC, Brunengraber H, Clish CB, DeBerardinis RJ, Feron O, Frezza C, Ghesquiere B, (2015). A roadmap for interpreting (13)C metabolite labeling patterns from cells. Curr Opin Biotechnol 34, 189–201. 10.1016/j.copbio.2015.02.003. 25731751 PMC4552607

[2] Jang C, Chen L, and Rabinowitz JD (2018). Metabolomics and Isotope Tracing. Cell 173, 822–837. 10.1016/j.cell.2018.03.055. 29727671 PMC6034115

[3] Kim IY, Suh SH, Lee IK, and Wolfe RR (2016). Applications of stable, nonradioactive isotope tracers in in vivo human metabolic research. Exp Mol Med 48, e203. 10.1038/emm.2015.97. 26795236 PMC4686699

[4] Sunny NE, Parks EJ, Browning JD, and Burgess SC (2011). Excessive hepatic mitochondrial TCA cycle and gluconeogenesis in humans with nonalcoholic fatty liver disease. Cell Metab 14, 804–810. 10.1016/j.cmet.2011.11.004. 22152305 PMC3658280

[5] Shulman GI, Rothman DL, Jue T, Stein P, DeFronzo RA, and Shulman RG (1990). Quantitation of muscle glycogen synthesis in normal subjects and subjects with non-insulin-dependent diabetes by 13C nuclear magnetic resonance spectroscopy. N Engl J Med 322, 223–228. 10.1056/NEJM199001253220403. 2403659

[6] Wolfe RR, and Peters EJ (1987). Lipolytic response to glucose infusion in human subjects. Am J Physiol 252, E218–223. 10.1152/ajpendo.1987.252.2.E218. 3826340

[7] Petersen KF, Befroy D, Dufour S, Dziura J, Ariyan C, Rothman DL, DiPietro L, Cline GW, and Shulman GI (2003). Mitochondrial dysfunction in the elderly: possible role in insulin resistance. Science 300, 1140–1142. 10.1126/science.1082889. 12750520 PMC3004429

[8] Fan TW, Lane AN, Higashi RM, Farag MA, Gao H, Bousamra M, and Miller DM (2009). Altered regulation of metabolic pathways in human lung cancer discerned by (13)C stable isotope-resolved metabolomics (SIRM). Mol Cancer 8, 41. 10.1186/1476-4598-8-41. 19558692 PMC2717907

[9] Ghergurovich JM, Lang JD, Levin MK, Briones N, Facista SJ, Mueller C, Cowan AJ, McBride MJ, Rodriguez ESR, Killian A, (2021). Local production of lactate, ribose phosphate, and amino acids within human triple-negative breast cancer. Med (N Y) 2, 736–754. 10.1016/j.medj.2021.03.009. PMC8248508 34223403

[10] Sellers K, Fox MP, Bousamra M 2nd, Slone SP, Higashi RM, Miller DM, Wang Y, Yan J, Yuneva MO, Deshpande R, (2015). Pyruvate carboxylase is critical for non-small-cell lung cancer proliferation. J Clin Invest 125, 687–698. 10.1172/JCI72873. 25607840 PMC4319441

[11] Maher EA, Marin-Valencia I, Bachoo RM, Mashimo T, Raisanen J, Hatanpaa KJ, Jindal A, Jeffrey FM, Choi C, Madden C, (2012). Metabolism of [U-13 C]glucose in human brain tumors in vivo. NMR Biomed 25, 1234–1244. 10.1002/nbm.2794. 22419606 PMC3406255

[12] Faubert B, Li KY, Cai L, Hensley CT, Kim J, Zacharias LG, Yang C, Do QN, Doucette S, Burguete D, (2017). Lactate Metabolism in Human Lung Tumors. Cell 171, 358–371.e359. 10.1016/j.cell.2017.09.019. PMC5684706 28985563

[13] Johnston K, Pachnis P, Tasdogan A, Faubert B, Zacharias LG, Vu HS, Rodgers-Augustyniak L, Johnson A, Huang F, Ricciardo S, (2021). Isotope tracing reveals glycolysis and oxidative metabolism in childhood tumors of multiple histologies. Med (N Y) 2, 395–410. 10.1016/j.medj.2021.01.002. PMC8045768 33860280

[14] Jeffrey FM, Rajagopal A, Malloy CR, and Sherry AD (1991). 13C-NMR: a simple yet comprehensive method for analysis of intermediary metabolism. Trends Biochem Sci 16, 5–10. 10.1016/0968-0004(91)90004-f. 2053137

[15] Malloy CR, Jones JG, Jeffrey FM, Jessen ME, and Sherry AD (1996). Contribution of various substrates to total citric acid cycle flux and anaplerosis as determined by 13C isotopomer analysis and O2 consumption in the heart. MAGMA 4, 35–46. 10.1007/BF01759778. 8774000

[16] Jeffrey FM, Roach JS, Storey CJ, Sherry AD, and Malloy CR (2002). 13C isotopomer analysis of glutamate by tandem mass spectrometry. Anal Biochem 300, 192–205. 10.1006/abio.2001.5457. 11779111

[17] Choi J, Grossbach MT, and Antoniewicz MR (2012). Measuring complete isotopomer distribution of aspartate using gas chromatography/tandem mass spectrometry. Anal Chem 84, 4628–4632. 10.1021/ac300611n. 22510303

[18] Noronha SB, Yeh HJ, Spande TF, and Shiloach J (2000). Investigation of the TCA cycle and the glyoxylate shunt in Escherichia coli BL21 and JM109 using (13)C-NMR/MS. Biotechnol Bioeng 68, 316–327. 10745200

[19] Kappelmann J, Klein B, Geilenkirchen P, and Noack S (2017). Comprehensive and accurate tracking of carbon origin of LC-tandem mass spectrometry collisional fragments for 13C-MFA. Analytical and Bioanalytical Chemistry 409, 2309–2326. 10.1007/s00216-016-0174-9. 28116490 PMC5477699

[20] Alves TC, Pongratz RL, Zhao X, Yarborough O, Sereda S, Shirihai O, Cline GW, Mason G, and Kibbey RG (2015). Integrated, Step-Wise, Mass-Isotopomeric Flux Analysis of the TCA Cycle. Cell Metab 22, 936–947. 10.1016/j.cmet.2015.08.021. 26411341 PMC4635072

[21] Liu H, Lam L, and Dasgupta PK (2011). Expanding the linear dynamic range for multiple reaction monitoring in quantitative liquid chromatography-tandem mass spectrometry utilizing natural isotopologue transitions. Talanta 87, 307–310. 10.1016/j.talanta.2011.09.063. 22099684

[22] Wei AAJ, Joshi A, Chen Y, and McIndoe JS (2020). Strategies for avoiding saturation effects in ESI-MS. International Journal of Mass Spectrometry 450, 116306.

[23] Cheng T, Sudderth J, Yang C, Mullen AR, Jin ES, Mates JM, and DeBerardinis RJ (2011). Pyruvate carboxylase is required for glutamine-independent growth of tumor cells. Proc Natl Acad Sci U S A 108, 8674–8679. 10.1073/pnas.1016627108. 21555572 PMC3102381

[24] Yang C, Harrison C, Jin ES, Chuang DT, Sherry AD, Malloy CR, Merritt ME, and DeBerardinis RJ (2014). Simultaneous steady-state and dynamic 13C NMR can differentiate alternative routes of pyruvate metabolism in living cancer cells. J Biol Chem 289, 6212–6224. 10.1074/jbc.M113.543637. 24415759 PMC3937686

[25] Pachnis P, Wu Z, Faubert B, Tasdogan A, Gu W, Shelton S, Solmonson A, Rao AD, Kaushik AK, Rogers TJ, Ubellacker JM, LaVigne CA, Yang C, Ko B, Ramesh V, Sudderth J, Zacharias LG, Martin-Sandoval MS, Do D, Mathews TP, Zhao Z, Miishra P, Morrison SJ, DeBerardinis RJ. (2022). In vivo isotope tracing reveals a requirement for the electron transport chain in glucose and glutamine metabolism by tumors. Science Advances In press. 10.1126/sciadv.abn9550 PMC9432826 36044570

[26] Davidson SM, Papagiannakopoulos T, Olenchock BA, Heyman JE, Keibler MA, Luengo A, Bauer MR, Jha AK, O’Brien JP, Pierce KA, (2016). Environment Impacts the Metabolic Dependencies of Ras-Driven Non-Small Cell Lung Cancer. Cell Metab 23, 517–528. 10.1016/j.cmet.2016.01.007. 26853747 PMC4785096

[27] Hensley CT, Faubert B, Yuan Q, Lev-Cohain N, Jin E, Kim J, Jiang L, Ko B, Skelton R, Loudat L, (2016). Metabolic Heterogeneity in Human Lung Tumors. Cell 164, 681–694. 10.1016/j.cell.2015.12.034. 26853473 PMC4752889

[28] Duan L, Cooper DE, Scheidemantle G, Locasale JW, Kirsch DG, and Liu X (2022). (13)C tracer analysis suggests extensive recycling of endogenous CO2 in vivo. Cancer Metab 10, 11. 10.1186/s40170-022-00287-8. 35799202 PMC9264524

[29] Courtney KD, Bezwada D, Mashimo T, Pichumani K, Vemireddy V, Funk AM, Wimberly J, McNeil SS, Kapur P, Lotan Y, (2018). Isotope Tracing of Human Clear Cell Renal Cell Carcinomas Demonstrates Suppressed Glucose Oxidation In Vivo. Cell Metab 28, 793–800 e792. 10.1016/j.cmet.2018.07.020. PMC6221993 30146487

[30] Mashimo T, Pichumani K, Vemireddy V, Hatanpaa KJ, Singh DK, Sirasanagandla S, Nannepaga S, Piccirillo SG, Kovacs Z, Foong C, (2014). Acetate is a bioenergetic substrate for human glioblastoma and brain metastases. Cell 159, 1603–1614. 10.1016/j.cell.2014.11.025. 25525878 PMC4374602

[31] Faubert B, Tasdogan A, Morrison SJ, Mathews TP, and DeBerardinis RJ (2021). Stable isotope tracing to assess tumor metabolism in vivo. Nat Protoc 16, 5123–5145. 10.1038/s41596-021-00605-2. 34535790 PMC9274147

[32] Wise DR, DeBerardinis RJ, Mancuso A, Sayed N, Zhang XY, Pfeiffer HK, Nissim I, Daikhin E, Yudkoff M, McMahon SB, and Thompson CB (2008). Myc regulates a transcriptional program that stimulates mitochondrial glutaminolysis and leads to glutamine addiction. Proc Natl Acad Sci U S A 105, 18782–18787. 10.1073/pnas.0810199105. PMC2596212 19033189

[33] Sanjana NE, Shalem O, and Zhang F (2014). Improved vectors and genome-wide libraries for CRISPR screening. Nat Methods 11, 783–784. 10.1038/nmeth.3047. 25075903 PMC4486245

[34] Ramaswamy V, Hooker JW, Withers RS, Nast RE, Brey WW, and Edison AS (2013). Development of a (1)(3)C-optimized 1.5-mm high temperature superconducting NMR probe. J Magn Reson 235, 58–65. 10.1016/j.jmr.2013.07.012. 23969086 PMC3785096

[35] Friedman J, Hastie T, and Tibshirani R (2010). Regularization Paths for Generalized Linear Models via Coordinate Descent. J Stat Softw 33, 1–22. 20808728 PMC2929880

[36] Lawson CL, Hanson RJ, Society for I, and Applied M (1995). Solving least squares problems.

[37] Katharine M Mullen I.H.M.v.S.e. (2012). nnls: The Lawson-Hanson algorithm for non-negative least squares (NNLS). .

[38] Borchers HW (2021). pracma: Practical Numerical Math Functions Release *2.3.3 ed*.

