# Supplementary material for: Comprehensive isotopomer analysis of glutamate and aspartate in small tissue samples
Source: Cell Metab. Author manuscript; Available in PMC 2023 Dec 20. (PMC10732579; doi:10.1016/j.cmet.2023.07.013)
Supplement: 2 — Supplemental Data I: MRM methods for glutamate and aspartate isotopomer analysis. Related to Figure 1 and STAR Methods. [file NIHMS1923036-supplement-2.pdf]

Supplemental Figure 1

A

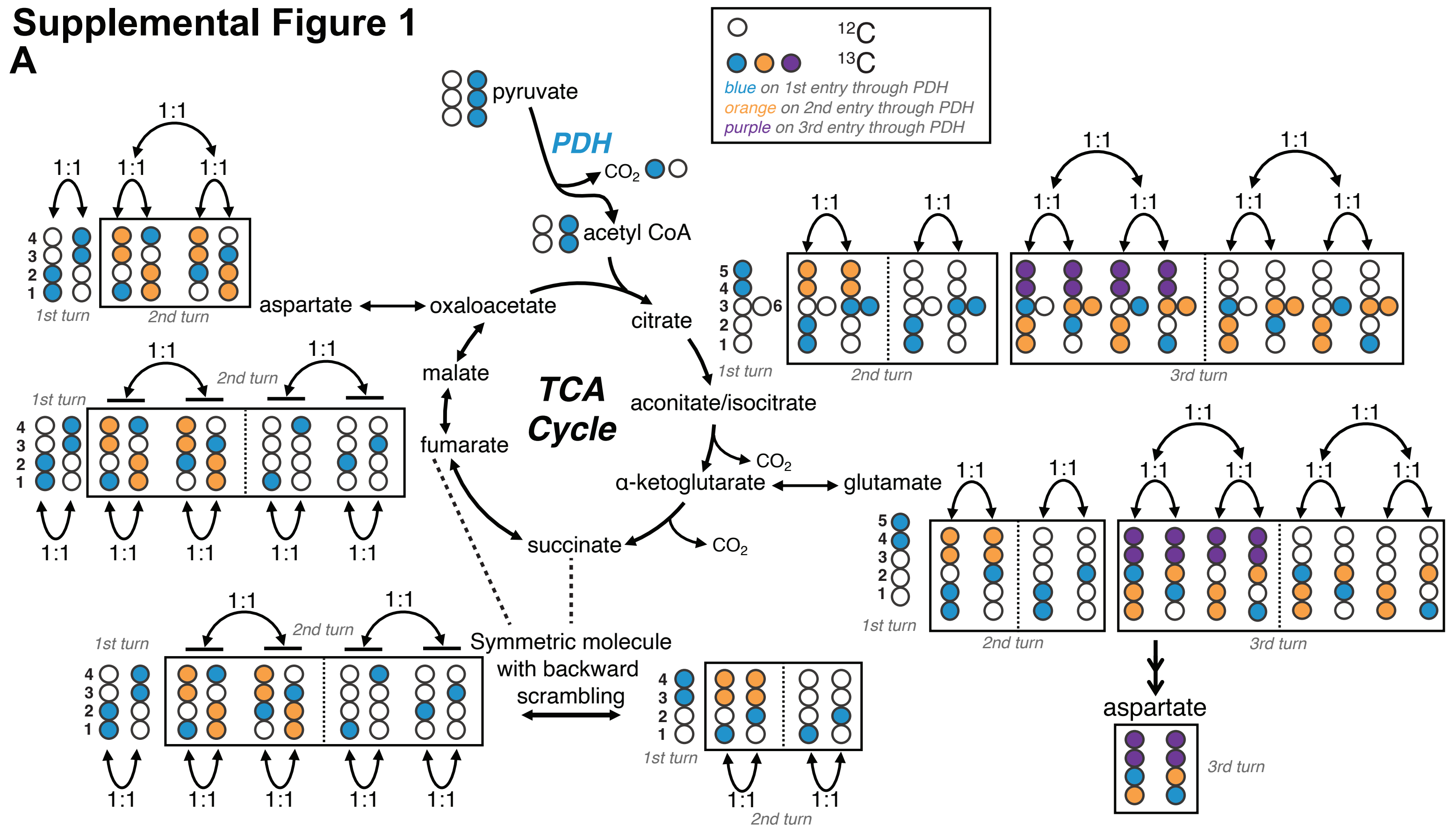

B

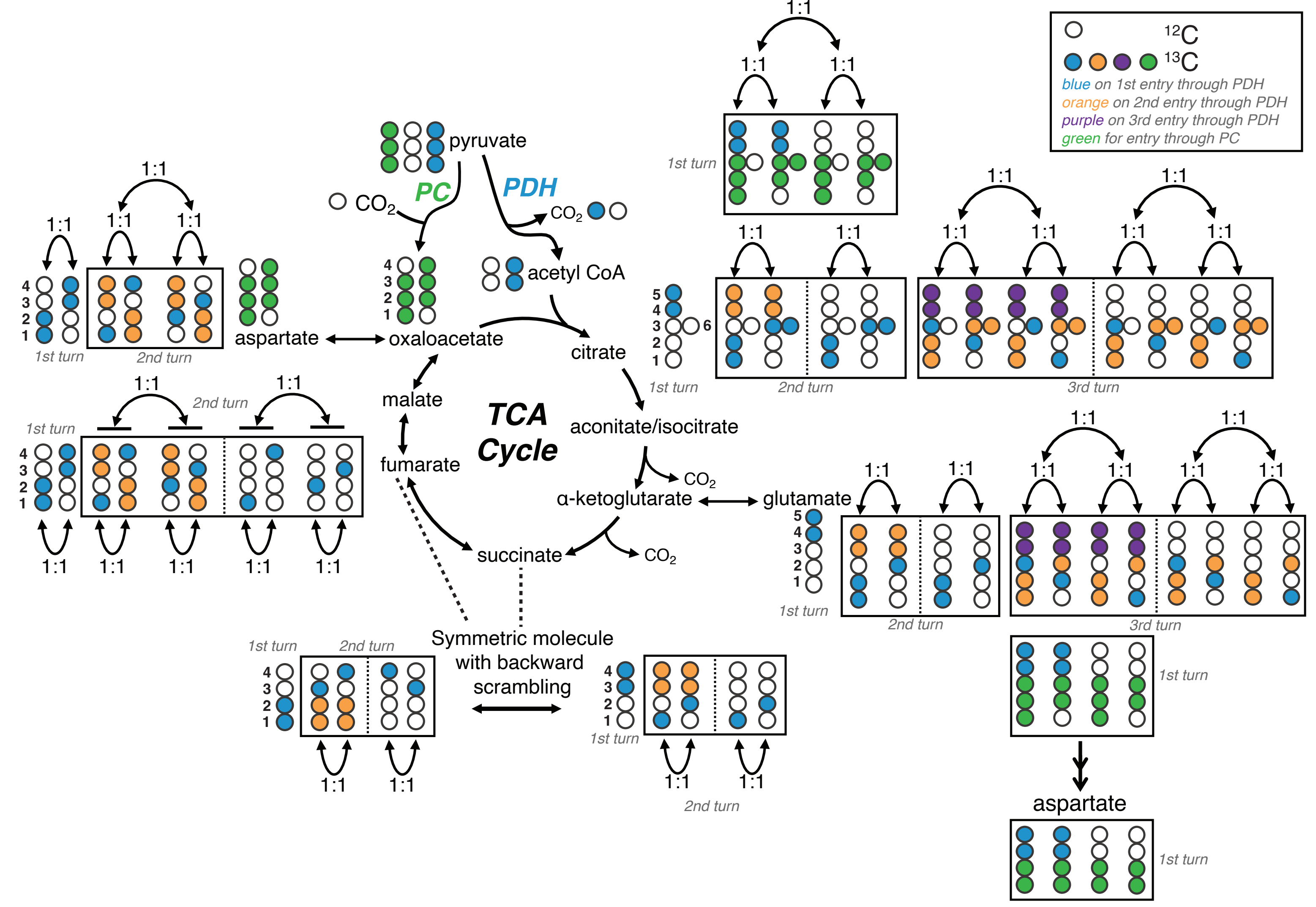

Supplemental Figure 2

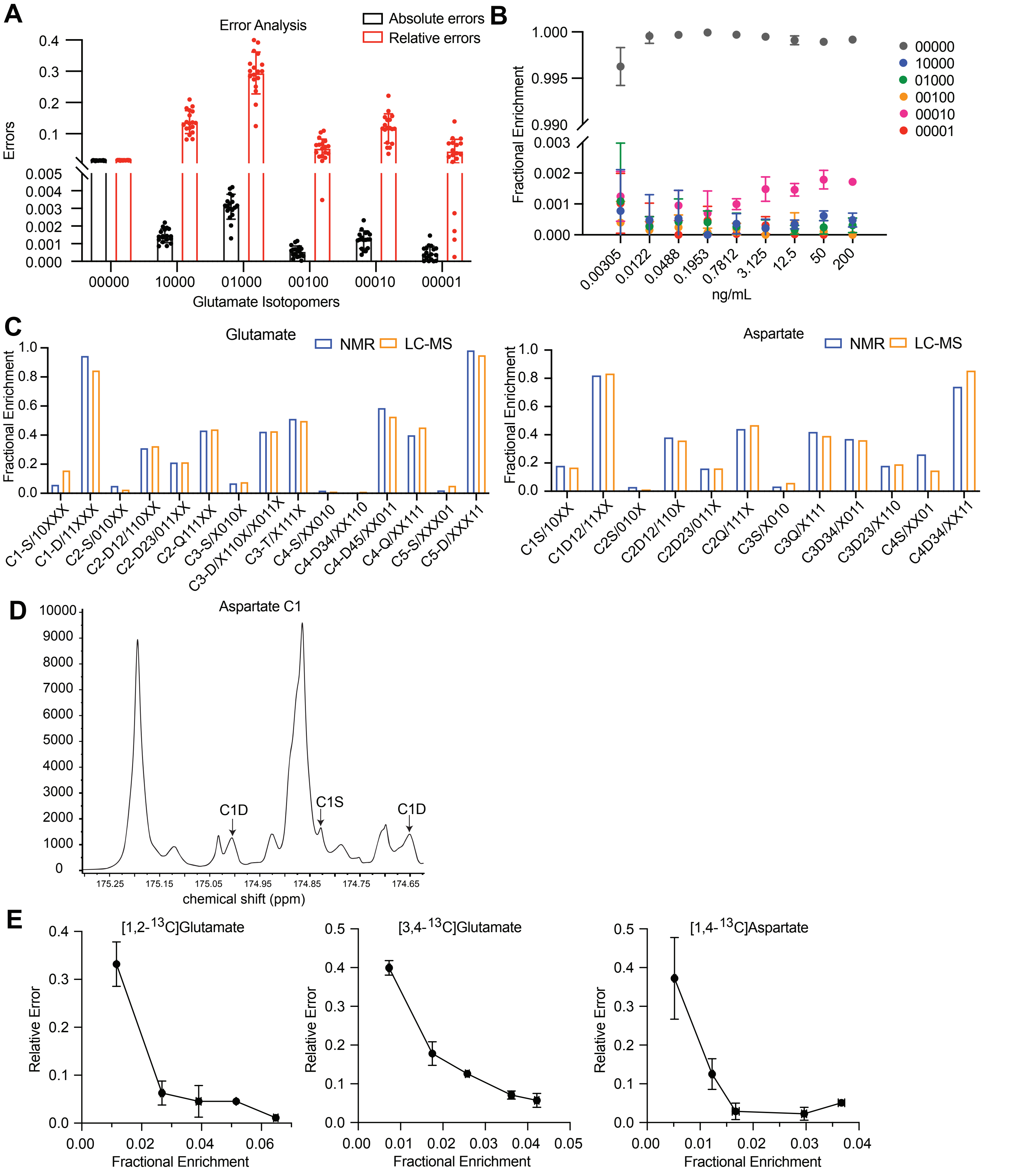

Supplemental Figure 3

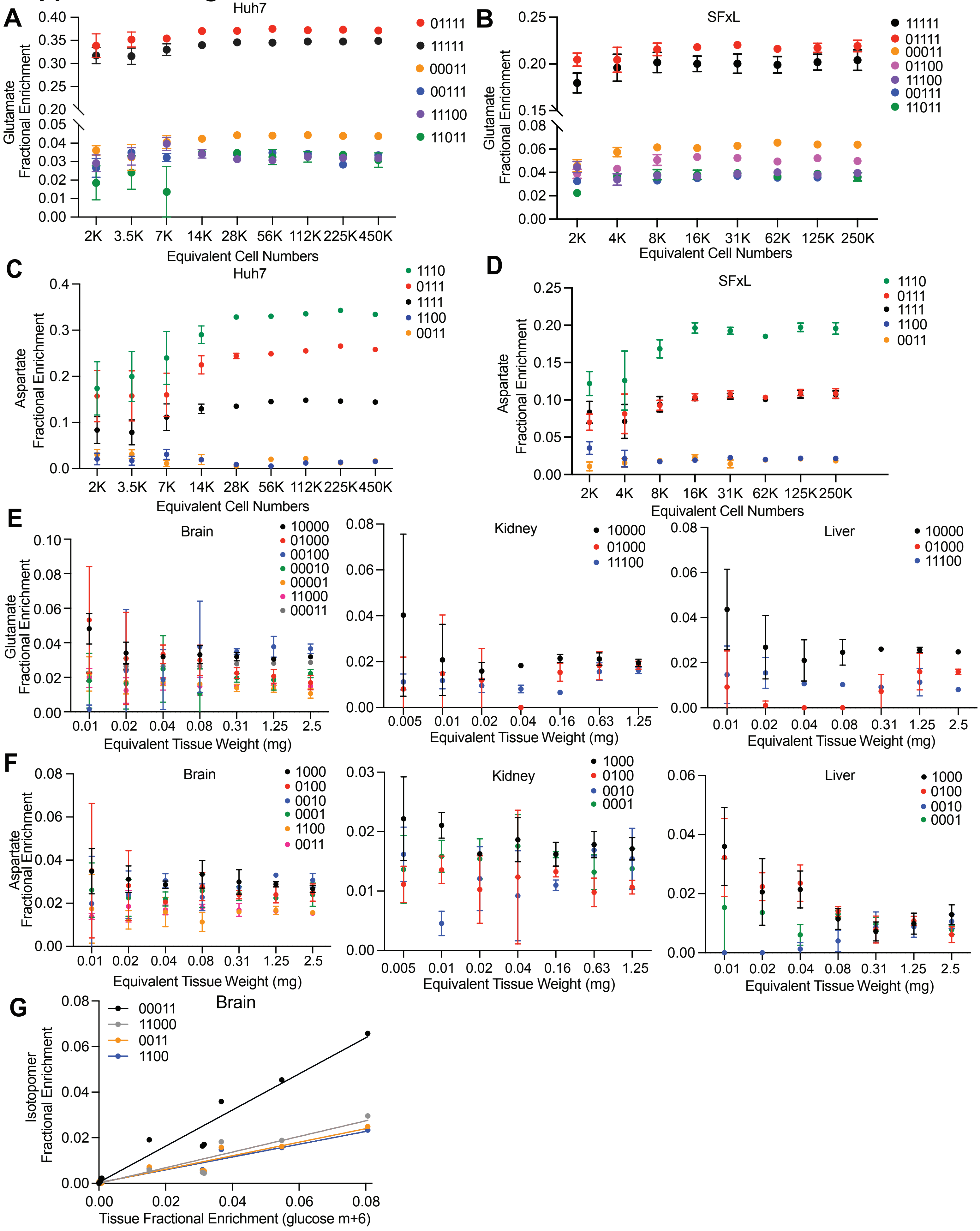

Supplemental Figure 4

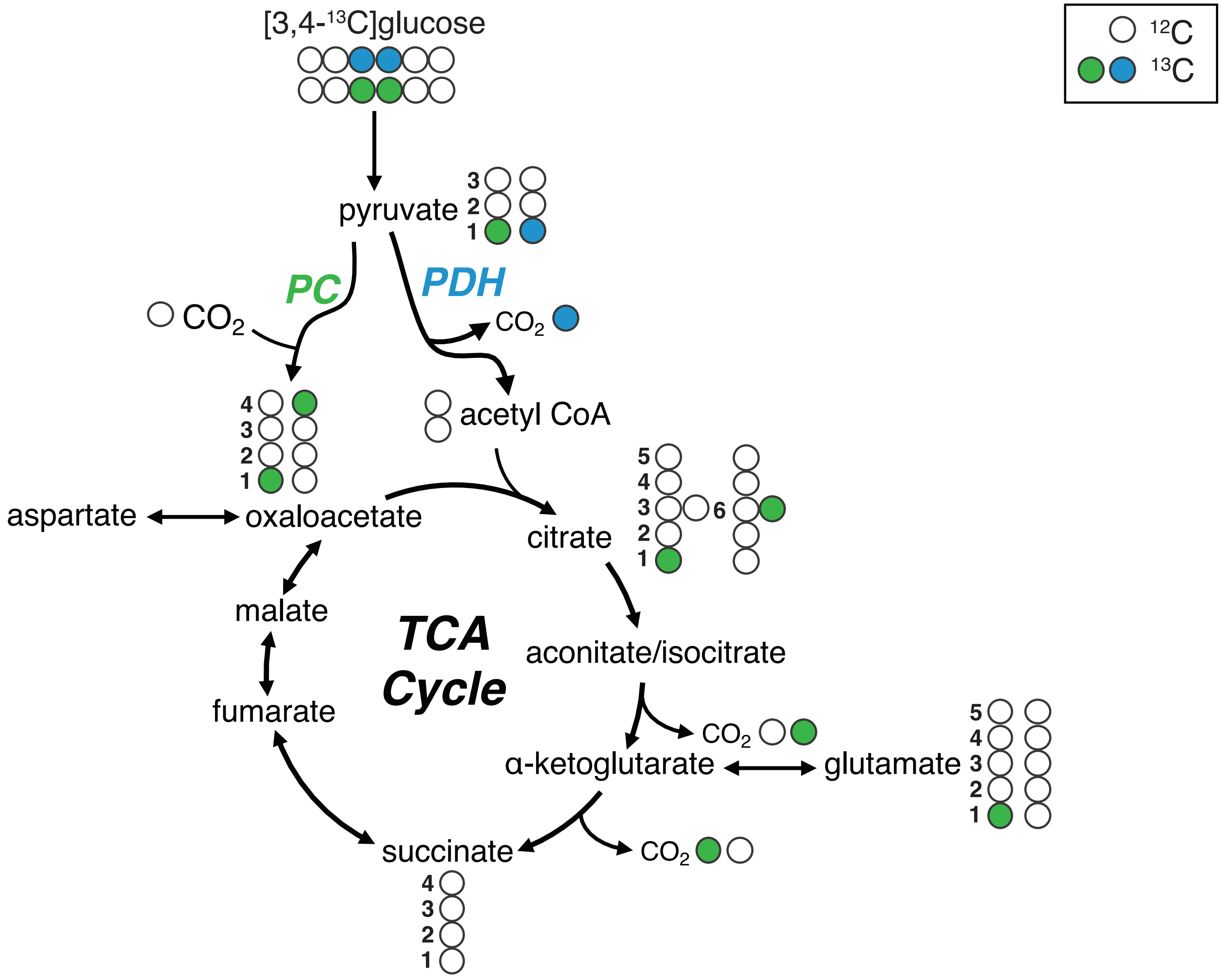

Supplemental Figure 5

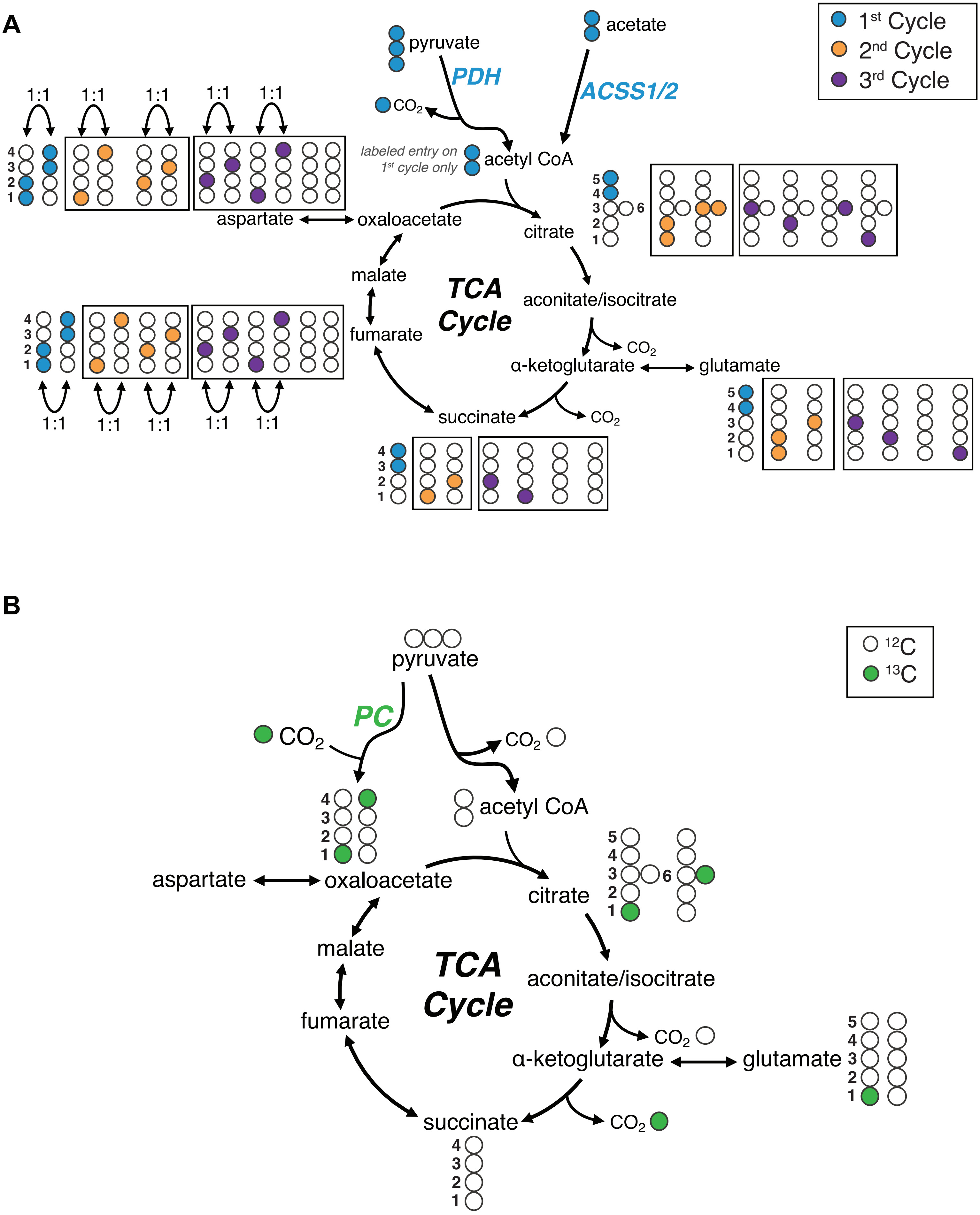

Supplemental Figure 6

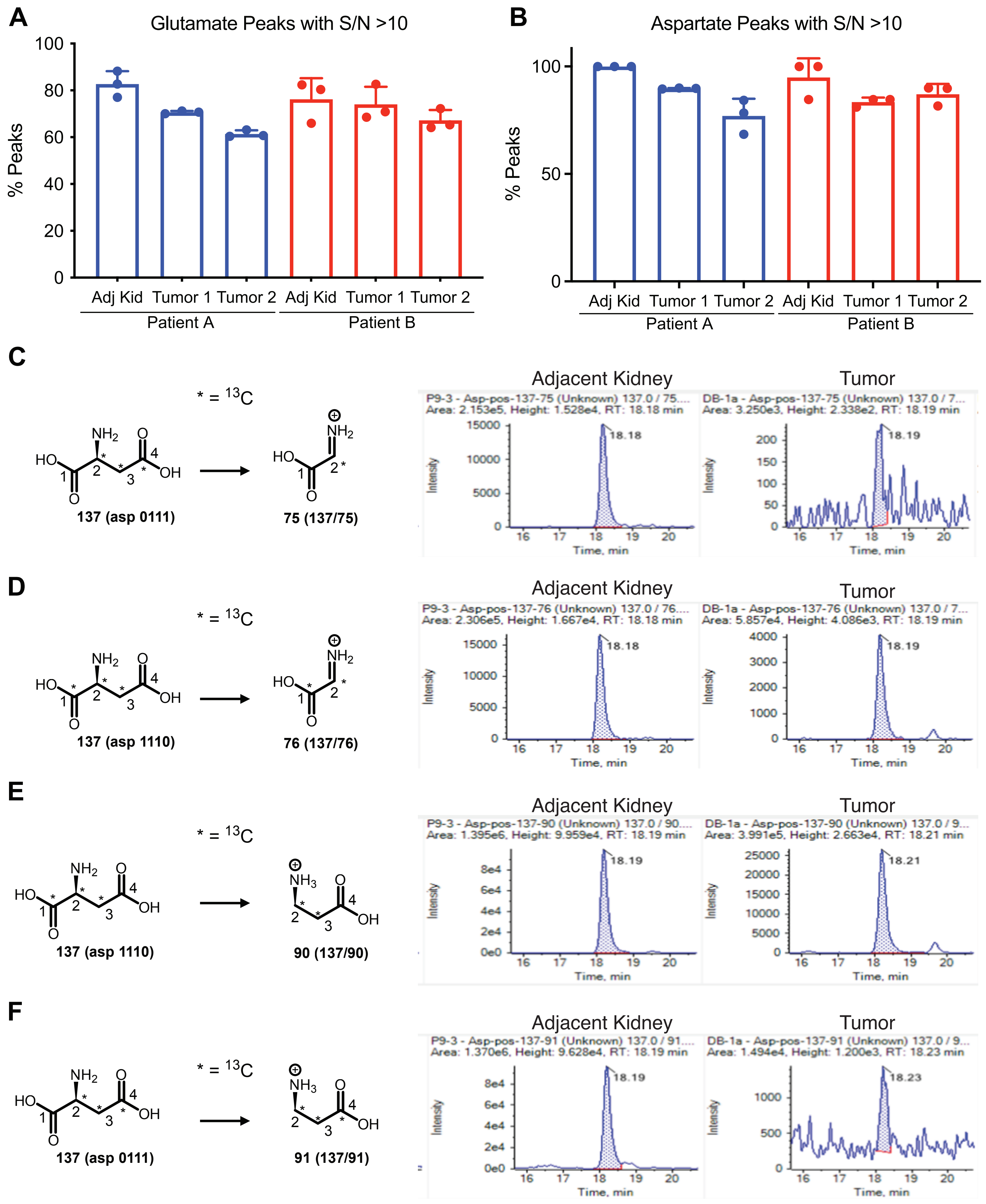

SUPPLEMENTAL FIGURE LEGENDS

Figure S1. **Schematic of isotopomer labeling from [U-^{13}C]pyruvate, related to Figure 1.** (**A**) Isotopomer schematic where PDH is active and PC is inactive. White circles indicate ^{12}C, while colored circles indicate ^{13}C. In citrate and glutamate, blue circles indicate carbons entering as acetyl-CoA from PDH on the first cycle, orange circles indicate carbons entering as acetyl-CoA from PDH on the second cycle, and purple circles indicate entering carbons entering as acetyl-CoA from PDH on the third cycle. In the four-carbon intermediates succinate, fumarate, malate, and aspartate, only first cycle (blue) and second cycle (orange) carbons are indicated. Succinate and fumarate (bottom of illustration) generate symmetrical isotopomers due to the symmetric nature of these metabolites. These symmetrical isotopomers are indicated with 1:1 arrows, and they give rise to additional corresponding isotopomers in subsequent steps of the cycle. This ultimately produces symmetric isotopomer pairs in aspartate with equal distribution in 1000/0001, 0100/0010, 1100/0011, and 1110/0111/1011/1101. The symmetric isotopomer labeling in oxaloacetate leads to multiple isotopomers in citrate and glutamate. The expected patterns occur in the same 1:1 ratios as oxaloacetate, and are indicated on citrate and glutamate by 1:1 arrows. Aspartate-1111 can arise from glutamate 11111 or 01111. The distribution of aspartate m+1 is discussed in Supplemental Figure 3A. (**B**) Isotopomer schematic where both PDH and PC are active. PC produces a new isotopomer of oxaloacetate (1110, green), which can then be converted to 0111 after equilibration with symmetric TCA cycle intermediates and eventually producing malate, oxaloacetate, and aspartate 1110/0111. The symmetrical isotopomers are indicated with 1:1 arrows. The large impact of PC on 1110 and 0111 means that these isotopomers can exceed 1011 and 1101; note that in Panel A, where PC is inactive, 1110, 0111, 1011 and 1101 are all present at equivalent abundances. Therefore the excess of 1110 and 0111 indicates the presence of PC activity.

Figure S2. Additional error analysis, related to Figure 2. (**A**) Positional error analysis of glutamate from Figure 1G with a naturally occurring glutamate standard. Absolute errors are less than 0.3% and relative errors are within 30% when the ^{13}C enrichment is as low as 1.1% (18 technical replicates per condition). (**B**) Glutamate isotopomer distribution after correction for natural abundance isotopes. Data are from the same non-corrected analysis shown in

Figure 1G (3 technical replicates). (**C**) ^{13}C NMR data in a 900 MHz instrument also provides largely consistent isotopomer distributions between NMR and LC-MS. (**D**) Aspartate C1 analysis from NMR. The inconsistency of aspartate C1 analysis between NMR and LC-MS is likely related to the poorly resolved aspartate C1 multiplet by NMR. (**E**) Relative errors of [1,2-^{13}C]glutamate, [3,4-^{13}C]glutamate, and [1,4-^{13}C]aspartate at increasing fractional enrichments (3 technical replicates).

Figure S3. Limitation of detection in cells and tissues, related to Figure 1 and 2. (**A-B**) Extracts of (A) 1.8M Huh7 cells and (B) extracts of 0.9M SFxL cells traced with [U-^{13}C]glucose. Extracts were serially diluted and analyzed for glutamate enrichments (3 biological replicates). (**C-D**) Extracts of (C) 1.8M Huh7 cells and (D) extracts of 0.9M SFxL cells traced with [U-^{13}C]glucose. Extracts were serially diluted and analyzed for aspartate enrichments (3 biological replicates). (**E-F**) [U-^{13}C]glutamine infused mouse brain, kidney, and liver tissues. Samples were serially diluted and analyzed for (E) glutamate and (F) aspartate isotopomers (3 biological replicates). (**G**) Isotopomer fractional enrichment compared to [U-^{13}C]glucose labeling in mouse brain tissue after infusion with [U-^{13}C]glucose.

Figure S4. Schematic of isotopomer labeling from [3,4-^{13}C]glucose, related to Figure 3. [3,4-^{13}C]glucose is converted to [1-^{13}C]pyruvate through glycolysis and enters into the TCA cycle through PDH or PC. If [1-^{13}C]pyruvate enters the TCA cycle through PDH, the ^{13}C at the C1 position will become $^{13}CO_2$, resulting in unlabeled acetyl-CoA. However, if [1-^{13}C]pyruvate enters the TCA cycle through PC, then the labeled carbon at C1 is retained as [1-^{13}C]oxaloacetate and later [4-^{13}C]oxaloacetate through symmetrization. [1-^{13}C] and [4-^{13}C]oxaloacetate become [1-^{13}C] and [6-^{13}C]citrate, and eventually [1-^{13}C]α-ketoglutarate and [1-^{13}C]glutamate. When [1-^{13}C]α-ketoglutarate is converted to succinate via succinyl-CoA, the remaining ^{13}C is released as $^{13}CO_2$ and subsequently produces unlabeled four carbon intermediates.

Figure S5. Schematic of M+1 isotopomer labeling, related to Figure 4. (**A**) Isotopomer schematic where PDH is active and PC is inactive. Equal distribution of both interior isotopomers (0100/0010) and exterior isotopomers (1000/0001) are generated in aspartate and other four-carbon intermediates due to backward scrambling in fumarate. [1,2-

^{13}C]acetyl-CoA is shown entering only on the first cycle for simplicity, and can arise from [U-^{13}C]pyruvate or [U-^{13}C]acetate. (**B**) Isotopomer schematic where both PDH and PC are active with an unlabeled source of pyruvate. $^{13}CO_2$ enters the TCA cycle through PC and it is converted to [4-^{13}C]oxaloacetate and [1-^{13}C]oxaloacetate, the latter arising through fumarate/succinate scrambling. [1-^{13}C] and [4-^{13}C]oxaloacetate will become [1-^{13}C] and [6-^{13}C]citrate, and eventually [1-^{13}C]α-ketoglutarate and [1-^{13}C]glutamate. When [1-^{13}C]α-ketoglutarate is converted to succinate via succinyl-CoA, the remaining ^{13}C is released as $^{13}CO_2$ and subsequently produces unlabeled four-carbon intermediates. Therefore, only [1-^{13}C] and [4-^{13}C]oxaloacetate (and [1-^{13}C] and [4-^{13}C]aspartate) are generated when the labeled carbon source is $^{13}CO_2$ incorporation from PC. Note that other carboxylase reactions giving rise to TCA cycle intermediates also result in labeling of external rather than internal carbons.

Figure S6. Sensitivity analysis of LC-MS/MS isotopomer method, related to Figure 5. (**A**-**B**) Summary of signal to noise ratios for HLRCC patient samples in glutamate and aspartate, respectively (3 biological replicates). (**C**-**F**) Representative chromatograms of peaks related to aspartate 1110 in adjacent kidney and tumor samples. Panels C and D reflect ion pairs that report labeling at C1 and C2 (137/75 and 137/76), which provide information about aspartate 0111 and 1110, respectively. Panels E and F reflect ion pairs that report labeling at C2, C3, and C4 (137/90 and 137/91), which provide information about aspartate 1110 and 0111, respectively.
